# Programming sequential deployment of origami via kinematic transition fronts

**DOI:** 10.1073/pnas.2615468123

**Published:** 2026-09-09

**Authors:** Rinki Imada, Tomohiro Tachi

**Affiliations:** ^a^ Department of Space Flight Systems, Institute of Space and Astronautical Science, Japan Aerospace Exploration Agency, Sagamihara-shi, Kanagawa 252-5210, Japan; ^b^ Department of General Systems Studies, Graduate School of Arts and Sciences, The University of Tokyo, Meguro-ku, Tokyo 153-8902, Japan

**Keywords:** transition fronts, origami, sequential deployment, discrete dynamical systems, heteroclinic orbits

## Abstract

Propagating transition fronts, in which local interactions sequentially trigger state changes, are widely observed across natural, biological, and engineered systems. While such propagation has been engineered using energy-driven instabilities, front propagation governed purely by geometric constraints remains underexplored and lacks a general design framework. In particular, how to program sequential deployment in origami through such kinematic propagation remains an open challenge. Here, we develop a systematic design framework for kinematic transition fronts based on their correspondence with heteroclinic orbits in discrete dynamical systems. Focusing on strips of developable and flat-foldable degree-4 origami vertices, we show that asymmetric coupling between adjacent creases produces nonlinear recurrence relations whose composition generically gives rise to heteroclinic orbits connecting developed and flat-folded states, enabling domino-like sequential deployment. We further show that macroscopic shape can be programmed independently of propagation behavior by exploiting invariances in the recurrence relation, and illustrate the approach through a representative thick-panel origami prototype. These results enable programmable sequential deployment in origami via transition fronts, while also establishing a general framework for kinematic transition fronts in geometrically constrained systems.

## Significance

Propagating transition fronts—where local changes trigger sequential responses—are common in nature and engineered systems, but are typically driven by stored energy and instability. Here, we focus on designing propagation that arises purely from geometry, without energy barriers. Using origami as a model system, we establish a design principle based on interpreting geometric constraints as discrete dynamical systems, enabling domino-like deployment into prescribed planar curves with independent control of propagation behavior. Because deformation remains localized, these structures reduce the swept region compared with uniformly deforming origami such as the Miura-ori. The kinematic theory and qualitative experimental validation presented suggest routes to deployable structures that form complex shapes in confined spaces and to designing kinematic transition fronts in geometry-based metamaterials.

Domino-like transitions (i.e., propagating transition fronts), in which local interactions trigger state changes, are a ubiquitous propagation phenomenon across natural, biological, and engineered systems. Examples include the cascading release of mechanical instabilities (1, 2), calcium waves in biological tissues (3), perversion points in helical structures (4, 5), and localized flipping in a ribbon-spread deck of cards (6). In recent years, mechanical metamaterials have provided a platform for engineering such propagating transition fronts, sometimes referred to as “solitons” in the literature, by designing interactions between constituent elements (7–10). A representative example is the transition wave, where the release of stored elastic energy in bistable units triggers sequential switching in neighboring elements (11–14). Propagating transition fronts can also arise from geometric interactions, without relying on energy barriers, as a purely kinematic, nonlinear phenomenon. Such phenomena have been reported in systems such as the Kane-Lubensky chain (15) in the context of topological mechanics (16–20), where geometric constraints dictate the order and direction of transitions. These mechanisms are not limited to linkage models but can be realized in a broader class of systems governed by geometric compatibility. However, such kinematic transition fronts remain largely underexplored and lack a general design framework.

Origami provides a particularly compelling platform in this regard, as it offers a geometric principle for compactly storing large systems. While many canonical mechanisms, such as the Miura-ori, require globally coordinated actuation in which all creases must be driven simultaneously, connecting a compact stowed state and a deployed state via a propagating transition front would enable deployment through spatially localized, sequential actuation. This removes the need for global synchronization and facilitates scalable actuation strategies. Moreover, such sequential deployment can reduce the swept volume during actuation, since only a localized region undergoes large motion at each stage, which is critical in spatially confined environments. These features are advantageous for applications such as minimally invasive medical devices (e.g., catheters and endoscopes), inspection tools for confined infrastructures, and the installation of structures within existing built environments. It may also simplify the programming of folding sequences by embedding actuation order into the geometry, avoiding the need for crease-by-crease stimulus tuning in conventional self-folding approaches (21). However, despite these potential advantages, a general design framework for sequential deployment in origami remains lacking.

Here, we present a systematic design strategy for origami-based mechanisms that realize kinematic propagation of transition fronts. Specifically, we focus on a one-degree-of-freedom strip of connected degree-4 vertices, a building block in origami kinematics. Although origami structures composed of degree-4 vertices, such as the Miura-ori, have been extensively studied in the contexts of kinematics (22–25), discrete differential geometry (26–30), and engineering (31–34), the realization of propagating transition fronts in such systems has remained elusive and was previously considered unattainable (18). Recent studies, however, have suggested that this limitation can be overcome by modifying the connectivity of crease patterns (35). The degree-4 origami strip is a one-dimensional Maxwell lattice (17, 20, 36), in which specifying the folded state of the boundary vertex determines the states of subsequent vertices in the bulk (18, 35). Previous work has shown that, when the recurrence relation between adjacent cells is interpreted as a discrete dynamical system (35–41), heteroclinic orbits emerging in sufficiently nonlinear regimes give rise to propagating transition fronts (35). However, only a few specific examples have been reported, and a general design methodology has not yet been established; moreover, these findings have remained largely theoretical without experimental validation.

Building on the correspondence between heteroclinic orbits in discrete dynamical systems and the kinematic propagation of transition fronts, we design developable and flat-foldable degree-4 origami strips that deploy in a domino-like manner and qualitatively validate them using physical prototypes. We first focus on strips for which both the connectivity and the sector angles are periodic. Based on the asymmetry in the fold angles of adjacent creases at each vertex, we show that, when adjacent creases are used to connect vertices, heteroclinic orbits generically emerge over a broad parameter range, leading to domino-like deployment, as demonstrated through representative design examples. We then consider strips in which the sector angles are allowed to vary along the strip, while the connectivity remains periodic. We show that, by transforming the crease pattern under conditions that preserve the recurrence relation, the macroscopic shape of the developed state can be programmed independently while maintaining the propagation behavior. In addition, we implement a representative design as a thick-panel origami structure using a thickness-accommodation technique, and experimentally observe the sequential deployment in a 3D-printed prototype. These results not only enable the systematic design of sequential deployment in origami, but also provide a general framework for engineering kinematic transition fronts beyond origami, with potential applications in deployable structures, robotics, and metamaterials.

## Results

### Parameterization.

**Fig. 1.**
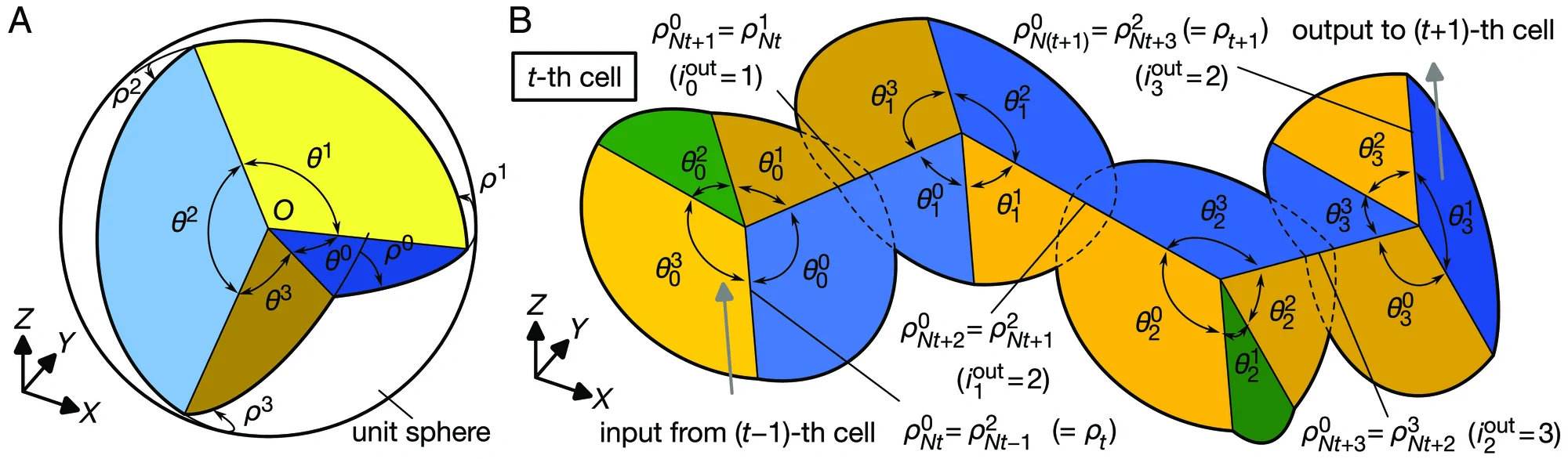
(*A*) Parameterization of the design and kinematics of a single degree-4 vertex by the sector angles ${\left(\theta^{i}\right)}_{i=0,1,2,3}$ and fold angles ${\left(\rho^{i}\right)}_{i=0,1,2,3}$. The sector angle $\theta^{i}$ denotes the angle between the *i*-th and (i+1)-th creases, and the fold angle $\rho^{i}$ denotes the exterior dihedral angle between the two facets sharing the *i*-th crease. A valley (mountain) fold corresponds to $\rho^{i}>0$ ($\rho^{i}<0$). A single degree-4 vertex can be represented as a spherical quadrilateral with fixed arc lengths ${\left(\theta^{i}\right)}_{i=0,1,2,3}$ by intersecting the crease pattern with a unit sphere centered at the vertex *O*, i.e., a spherical 4R mechanism. (*B*) Example of the unit-cell of a periodic degree-4 origami strip [$N=4,\left(i_{0}^{\text{out}},i_{1}^{\text{out}},i_{2}^{\text{out}},i_{3}^{\text{out}}\right)=\left(1,2,3,2\right)$].

We introduce a minimal parameterization of the crease pattern design and kinematics of periodic origami strips, following our previous work (35). A single degree-4 vertex is parameterized by labeling its creases counterclockwise as i=0,1,2,3, and introducing the sector angles $\left(\theta^{0},\theta^{1},\theta^{2},\theta^{3}\right)$ and the fold angles $\left(\rho^{0},\rho^{1},\rho^{2},\rho^{3}\right)$, where $\theta^{i}\in \left(0,\pi \right)$ and $\rho^{i}\in \left[-\pi ,\pi \right]$ (Fig. 1*A*). Imposing developability and flat-foldability leads to the constraints $\theta^{0}+\theta^{1}+\theta^{2}+\theta^{3}=2\pi$ and $\theta^{0}-\theta^{1}+\theta^{2}-\theta^{3}=0$, which are equivalent to $\theta^{2}=\pi -\theta^{0}$ and $\theta^{3}=\pi -\theta^{1}$. For a strip, we label the vertices by n=0,1,⋯ and denote the sector angles and fold angles at the *n*-th vertex by ${\left(\theta_{n}^{i}\right)}_{i=0,1,2,3}$ and ${\left(\rho_{n}^{i}\right)}_{i=0,1,2,3}$. When vertices are connected in order of *n*, each vertex shares creases with its neighbors, defining input and output creases. Here, we fix the input crease to i=0 and denote the output crease index at the *n*-th vertex by $i_{n}^{\text{out}}\in \left\{1,2,3\right\}$. While most previous studies have focused on the case where opposite creases are used to connect neighboring vertices ($i_{n}^{\text{out}}=2$) (18, 35), here we also allow the use of adjacent creases ($i_{n}^{\text{out}}=1,3$). The crease pattern of the strip is thus specified by ${\left({\left(\theta_{n}^{i}\right)}_{i=0,1}\right)}_{n=0,1,\cdots }$ and ${\left(i_{n}^{\text{out}}\right)}_{n=0,1,\cdots }$, where the latter fully specifies the connectivity of the strip. In this study, we first consider periodic degree-4 strips, defined as strips whose sector angles and output crease indices are both periodic, i.e., $\theta_{n+N}^{i}=\theta_{n}^{i}$ and $i_{n+N}^{\text{out}}=i_{n}^{\text{out}}$ ($N\in Z_{\geq 1}$). Owing to the periodicity, we define the *t*-th cell by *Nt*-th to (N(t+1)−1)-th vertices (t=0,1,⋯). Consequently, the crease pattern of a periodic, developable, and flat-foldable strip is fully characterized by the period *N*, the sector angles and output crease indices of a unit cell, ${\left({\left(\theta_{n}^{i}\right)}_{i=0,1}\right)}_{n=0,1,\cdots ,N-1}$ and ${\left(i_{n}^{\text{out}}\right)}_{n=0,\cdots ,N-1}$ (Fig. 1*B*). Although crease lengths are additional design parameters, they do not affect the resulting discrete dynamical system and are therefore omitted, except when specifying concrete examples later.

### Asymmetric Coupling of Adjacent Fold Angles.

The sequence of fold angles in the strip follows the kinematic relations for a developable and flat-foldable degree-4 vertex:

[1] $$\begin{array}{l} \begin{array}{ll} \rho^{1}= & -\;\text{sgn}(\rho^{0})\;\text{sgn}(\text{cos}\;\theta^{0}+\sigma \;\text{cos}\;\theta^{1})\\ & \times \;\text{arccos}\left(\frac{A\text{cos}\rho^{0}+B}{B\text{cos}\rho^{0}+A}\right), \end{array}\\ \begin{array}{ll} \rho^{2}\;= & \sigma \rho^{0},\\ \rho^{3}= & -\sigma \rho^{1},\\ \text{where} & A:=\text{cos}\;\theta^{0}\text{cos}\;\theta^{1}+\sigma ,\;B\;\text{sin}\;\theta^{0}\text{sin}\;\theta^{1}, \end{array}\\ \begin{array}{l} \begin{array}{ll} & \;\;\;\;\;\;\;\;\;\;\sigma :=\text{sgn}(\rho^{0})\;\text{sgn}(\rho^{2}). \end{array} \end{array} \end{array}$$

**Fig. 2.**
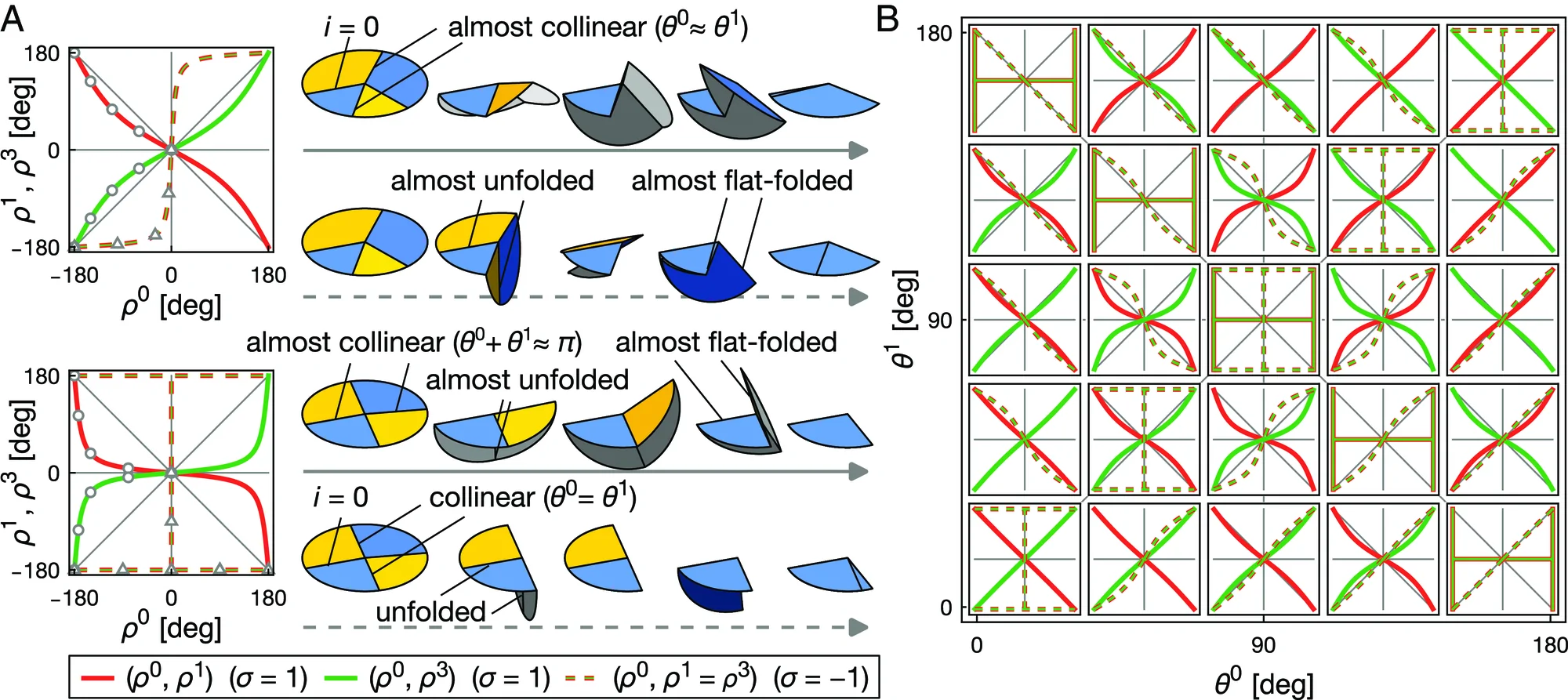
(*A*) Visualization of the relationship between adjacent fold angles and the folding motions for two representative examples [*Top*: $\left(\theta^{0},\theta^{1}\right)=\left(60^{{}^{\circ}},55^{{}^{\circ}}\right)$; *Bottom*: $\left(\theta^{0},\theta^{1}\right)=\left(85^{{}^{\circ}},85^{{}^{\circ}}\right)$]. In the *Left* panels, the folding modes corresponding to σ=1 (σ=−1) are shown as solid (dashed) curves in the parameter space. In the *Right* panels, folding motions from the developed state to the flat-folded state are visualized by sampling configurations along these curves, with circles (triangles) indicating σ=1 (σ=−1). Both examples have nearly collinear creases, causing the highly asymmetric folding behavior. In the *Bottom* example, a pair of creases (i=1,3) is exactly collinear ($\theta^{0}=\theta^{1}$), resulting in a decoupling between the two pairs (i=0,2) and (i=1,3) when σ=−1. In this case, the collinear pair can be flat-folded first without folding the other pair, corresponding to the vertical branch; upon reaching the flat-folded state $(\left(\rho^{0},\rho^{1}\right)=\left(0,-\pi \right)$ or (0,π)), the other pair (i=0,2) subsequently aligns and can also be flat-folded, corresponding to the horizontal branches in the *Left* panel. (*B*) Dependence of the adjacent fold-angle relations on $\left(\theta^{0},\theta^{1}\right)$. For representative sector angles, the relations are visualized as curves in the same format as in (*A*) and arranged over the $\left(\theta^{0},\theta^{1}\right)$-space, where $\theta^{0},\theta^{1}\in \left\{15^{{}^{\circ}},52.5^{{}^{\circ}},90^{{}^{\circ}},127.5^{{}^{\circ}},165^{{}^{\circ}}\right\}$. For panels along the diagonals $\theta^{0}=\theta^{1}$ ($\theta^{0}+\theta^{1}=\pi$), the curves for σ=−1 (σ=1) show the decoupling of the two pairs of opposite creases, similar to the behavior shown in the *Bottom* panels of (*A*). Except for the singular cases, each curve intersects the diagonal lines $\rho^{1}=\pm \rho^{0}$ and $\rho^{3}=\pm \rho^{0}$ only at (0,0), (±π,±π), or (±π,∓π) and the slope at these intersection points depends on $\left(\theta^{0},\theta^{1}\right)$. This reflects that the cosines $\cos\rho^{0}$ and $\cos\rho^{1}=\cos\rho^{3}$ are related by a symmetric fractional linear transformation, for which $\cos\rho^{0}=\pm 1$ are the only fixed points.

The sign parameter σ∈{−1,1} specifies whether the MV-assignments of opposite fold angles $\rho^{0}$ and $\rho^{2}$ are the same or different, which characterizes the folding mode. Note that Eq. **1** does not apply in the singular cases $\left(\theta^{0}=\theta^{1},\;\sigma =-1\right)$ and $\left(\theta^{0}+\theta^{1}=\pi ,\;\sigma =1\right)$. Eq. **1** can be derived from spherical trigonometry and is often expressed using alternative variables such as $\tan(\rho^{i}/2)$ (25, 42) (see *SI Appendix*, section 1 for a detailed derivation of Eq. **1**). From Eq. **1**, the input crease (i=0) and the opposite crease (i=2) evolve at the same rate; in contrast, the relations between the input crease and the adjacent creases (i=1,3) are nonlinear. Fig. 2 *A* and *B* illustrate the adjacent fold-angle relations and the corresponding folding motions for two representative values of $\left(\theta^{0},\theta^{1}\right)$, as well as their dependence on $\left(\theta^{0},\theta^{1}\right)$ [Movies S1 and S2, showing folding motions over a more densely sampled set of $\left(\theta^{0},\theta^{1}\right)$]. As shown in Fig. 2*A*, the adjacent creases exhibit asymmetric folding rates: For σ=1 (σ=−1), they evolve more slowly (more rapidly) than the input crease when $\rho^{0}$ increases from 0 to *π* (or −π), catching up near the flat-folded state. This asymmetry is quantified by the slopes $|d\rho^{1}/d\rho^{0}|=|d\rho^{3}/d\rho^{0}|$ evaluated at $\rho^{0}=0$ or $\rho^{0}=\pm \pi$, which are reciprocal to each other and given by $\sqrt{\left(A-B)/(A+B\right)}$ and its inverse, respectively. These quantities are known as the folding multiplier (26, 43–45), which has primarily been used to assess rigid foldability, but has not been interpreted as a measure of asymmetry. As these ratios approach 0 or *∞*, the asymmetry becomes increasingly pronounced. Geometrically, the singular cases ($\theta^{0}=\theta^{1}$, or $\theta^{0}+\theta^{1}=\pi$) correspond to crease patterns in which opposite creases (i=1 and 3, or i=0 and 2) become collinear in the developed state. As shown in the *Bottom* panel of Fig. 2*A*, when the opposite creases are collinear, they can fold flat without actuating the other creases, i.e., the two pairs of opposite creases become kinematically decoupled. The asymmetry becomes most pronounced when the opposite creases are nearly collinear so that the two pairs remain only marginally kinematically coupled. For a strip, the sequence of input fold angles, ${\left(\rho_{n}^{0}\right)}_{n=0,1,\cdots }$, is generated by successive applications of local maps $f_{n}$ to $\rho_{0}^{0}$, where each $f_{n}$ maps the *n*-th input fold angle $\rho_{n}^{0}$ to the output fold angle specified by the index $i_{n}^{\text{out}}$. By connecting vertices through adjacent creases, the imbalance between adjacent fold angles accumulates along the strip, providing the mechanism for domino-like deployment.

### Heteroclinic Orbits as a Design Principle for Domino-Like Deployment.

**Fig. 3.**
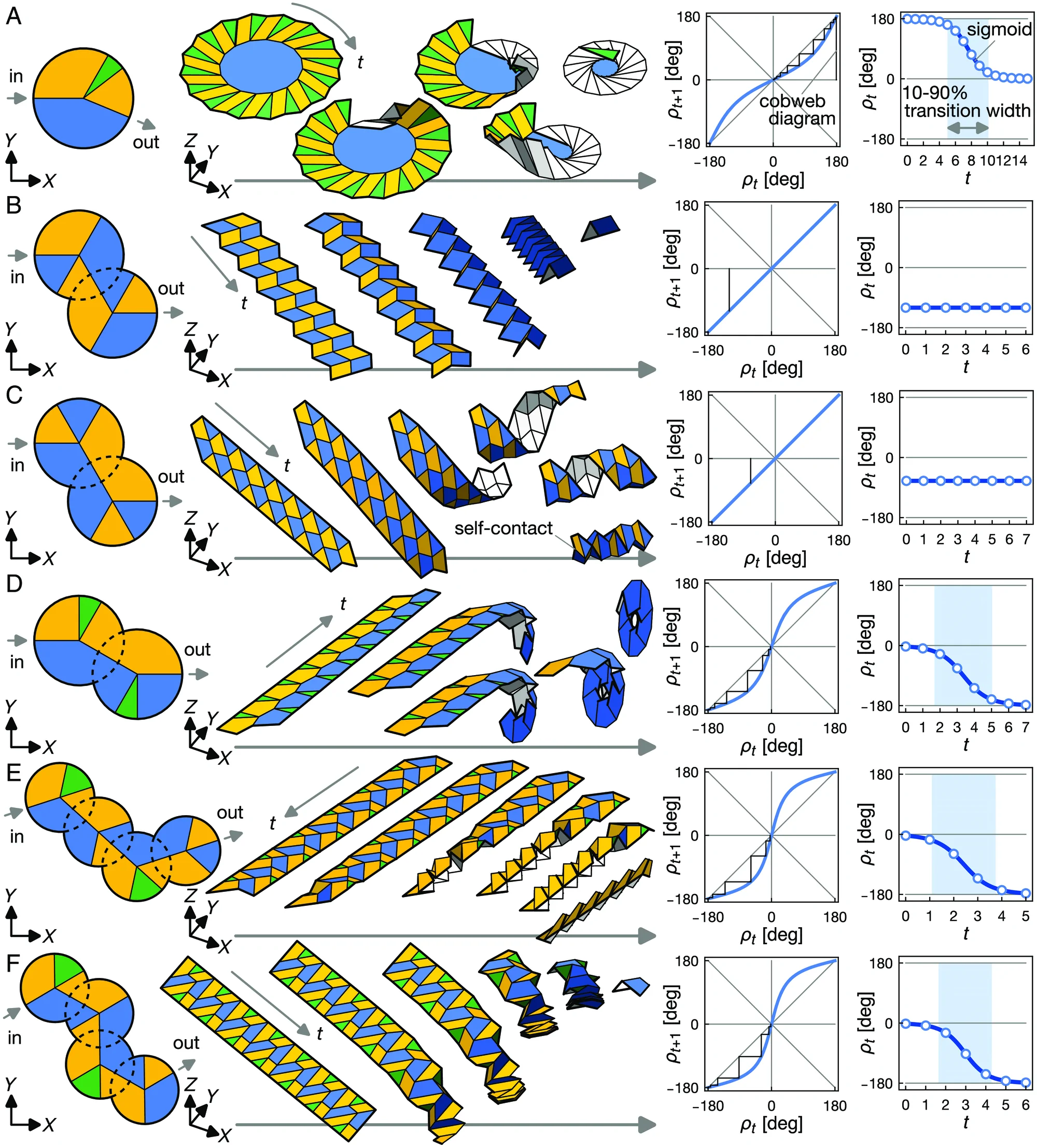
Examples of the periodic degree-4 strips, exhibiting the uniform or domino-like deployment. Each panel includes the unit-cell design, folding motion between a flat-folded state and a developed state, graph of $\rho_{t+1}=f\left(\rho_{t}\right)$ with a cobweb diagram for one intermediate folded state, and the plot of the input fold angles ${\left(\left(t,\rho_{t}\right)\right)}_{t=0,1\cdots }$ for that state. In the plots of the orbits, the underlying sigmoid function on which the orbits lie is shown, with its 10 to 90% transition width highlighted by shading. In visualizing folding motions, the crease lengths are specified, which do not affect the kinematic coupling behavior. (*A*) N=1 and $\left(\left(\theta_{0}^{0},\theta_{0}^{1}\right),i_{0}^{\text{out}},\sigma_{0}\right)=\left(\left(157.5^{{}^{\circ}},60^{{}^{\circ}}\right),1,1\right)$. (*B*) N=2 and ${\left(\left(\theta_{n}^{0},\theta_{n}^{1}\right),i_{n}^{\text{out}},\sigma_{n}\right)}_{n=0,1}=\left(\left(\left(60^{{}^{\circ}},60^{{}^{\circ}}\right),2,1\right),\left(\left(120^{{}^{\circ}},120^{{}^{\circ}}\right),2,1\right)\right)$. (*C*) N=2 and ${\left(\left(\theta_{n}^{0},\theta_{n}^{1}\right),i_{n}^{\text{out}},\sigma_{n}\right)}_{n=0,1}=\left(\left(\left(120^{{}^{\circ}},120^{{}^{\circ}}\right),1,1\right),\left(\left(120^{{}^{\circ}},60^{{}^{\circ}}\right),3,-1\right)\right)$. The folding motion stops before reaching the flat-folded state, because of the self-contact. (*D*) N=2 and ${\left(\left(\theta_{n}^{0},\theta_{n}^{1}\right),i_{n}^{\text{out}},\sigma_{n}\right)}_{n=0,1}=\left(\left(\left(150^{{}^{\circ}},90^{{}^{\circ}}\right),1,-1\right),\left(\left(90^{{}^{\circ}},30^{{}^{\circ}}\right),3,-1\right)\right)$. (*E*) N=4 and ${\left(\left(\theta_{n}^{0},\theta_{n}^{1}\right),i_{n}^{\text{out}},\sigma_{n}\right)}_{n=0,1,2,3}=\left(\left(\left(120^{{}^{\circ}},60^{{}^{\circ}}\right),1,-1\right),\left(\left(120^{{}^{\circ}},60^{{}^{\circ}}\right),2,-1\right),\left(\left(120^{{}^{\circ}},60^{{}^{\circ}}\right),3,-1\right),\left(\left(120^{{}^{\circ}},60^{{}^{\circ}}\right),2,-1\right)\right)$. (*F*) N=4 and ${\left(\left(\theta_{n}^{0},\theta_{n}^{1}\right),i_{n}^{\text{out}},\sigma_{n}\right)}_{n=0,1,2,3}=\left(\left(\left(120^{{}^{\circ}},60^{{}^{\circ}}\right),1,-1\right),\left(\left(60^{{}^{\circ}},60^{{}^{\circ}}\right),2,1\right),\left(\left(120^{{}^{\circ}},60^{{}^{\circ}}\right),3,-1\right),\left(\left(120^{{}^{\circ}},120^{{}^{\circ}}\right),2,1\right)\right)$.

For a *N*-periodic strip with $((\left(\theta_{n}^{0},\theta_{n}^{1}\right),$$i_{n}^{\text{out}},\sigma_{n}{))}_{n=0,\cdots ,N-1}$, we define the composite map $f:=f_{N-1}{}^{\circ}\cdots {}^{\circ}f_{0}$ which maps the input fold angle of one unit cell, $\rho_{t}:=\rho_{\mathrm{Nt}}^{0}$, to that of the next cell, $\rho_{t+1}$ (Fig. 1*B*). As the simplest example, when N=1 and $i_{0}^{\text{out}}=1$ or 3, the map *f* reduces to the relation between adjacent fold angles in Eq. **1**. The folding motion of the strip in this class is visualized in Fig. 3*A*, where the imbalance between input fold angle $\rho_{n}^{0}$ and output fold angle $\rho_{n}^{1}$ accumulates along the strip, resulting in domino-like deployment (see *SI Appendix*, section 2 for computational details; Movie S3). Here, by interpreting the cell index *t* as discrete time, the map *f* defines a discrete dynamical system in which the developed state (ρ=0) and the flat-folded state (ρ=π or −π) are fixed points. The fold angle sequence ${\left(\rho_{t}\right)}_{t=0,\cdots }$ observed during domino-like deployment (Fig. 3*A*, *Right* panel) exhibits a sharp transition from values near *π* to values approaching 0. Such behavior is consistent with a heteroclinic connection between these fixed points; accordingly, the fold angle sequence can be interpreted as a finite portion of an infinite heteroclinic orbit, i.e., a trajectory that approaches one fixed point as t→−∞ and the other as t→+∞. Notably, Eq. **1** implies that the cosines of adjacent fold angles are related by a symmetric fractional linear transformation. Such transformations are characterized, up to an overall scaling, by the ratio A/B, which therefore serves as the effective parameter of the map. Furthermore, the orbit ${\left(\rho_{t}\right)}_{t=0,\cdots }$ lies on the function of *t* defined by:

[2] $$\begin{array}{c} \begin{array}{cc} & 2\arctan\left(\left(\tan\frac{\rho_{0}}{2}\right)p^{t}\right),\\ \text{where}\;p & :=-\;\text{sgn}\left(\cos\theta^{0}+\sigma \cos\theta^{1}\right)\sqrt{\frac{A-B}{A+B}}. \end{array} \end{array}$$

That is, if we (formally) extend the exponent *t* in Eq. **2** to take real values, the discrete orbit coincides with the samples at integer *t* of the continuous function defined by Eq. **2** (see *SI Appendix*, section 3 for a detailed derivation of Eq. **2**). This function is a continuous sigmoid on R for p>0; for p<0, the same property holds after taking its absolute value. By examining the 10 to 90% transition width of this sigmoid function, |2log(tan(π/20))/logp|, one can estimate how many unit cells are required for the domain wall connecting the developed and flat-folded states. For example, in Fig. 3*A*, the transition width corresponds to approximately five unit cells. Although the above concerns the case of N=1, the class of symmetric fractional linear transformations is closed under composition, so that the composite map *f* retains the same functional form; thus, the same argument applies to N>1. Provided that the composite map *f* is not the identity (i.e., B≠0, where *B* denotes the parameter of the composite map *f*), the fixed points are universally given by $\cos\rho_{t}=\pm 1$, independent of the parameters, while their stability, as quantified by the slope at the fixed points, depends on the parameters (Fig. 2*B*). The degeneration to the identity arises, for example, when $i_{n}^{\text{out}}=2$ for all *n* (Fig. 3*B* and Movie S4), or when effects of imbalances for multiple adjacent-crease connections cancel each other within a unit cell due to specific choices of sector angles (Fig. 3*C* and Movie S5). In many developable and flat-foldable quadrilateral-mesh origami, such as the Miura-ori (Fig. 3*B*) and chickenwire pattern (Fig. 3*C*), the substrips fall into these degenerate cases; consequently, most origami-based mechanisms based on these patterns exhibit uniform deformation. However, except for these degenerate cases, domino-like deployment generically occurs when adjacent creases are used. In other words, for the present discrete dynamical system, as long as the map is not the identity, the system always possesses two fixed points at the same locations, connected by a heteroclinic orbit, and therefore does not exhibit more complex behaviors such as period-doubling bifurcations or chaos (39, 40). In addition, transition fronts are collective phenomena arising from sequences of vertices; therefore, their existence does not require individual vertices to lie near a geometric singularity with nearly collinear creases, which may be sensitive to perturbations in sector angles in practice. Fig. 3*D*–*F* present representative generic examples in which the output crease indices and sector angles do not lead to a degeneration of the map *f* to the identity (Movies S6–S8). Note that, in Fig. 3*D*–*F*, the sector angles exhibit geometric symmetries (e.g., 1) vertices in each unit cell have identical sector angles up to relabeling, and 2) mirror symmetry at individual vertices, such as $\theta_{n}^{0}=\theta_{n}^{1}$ or $\theta_{n}^{0}+\theta_{n}^{1}=\pi$); however, these do not in general imply a degeneration to the identity. Fig. 3*D* has the same output crease indices as Fig. 3*C*, but different sector angles and mode assignments. Fig. 3 *E* and *F* correspond to designs obtained by inserting additional vertices with adjacent-crease connections in the vertex-sequence of a Miura-ori strip (specifically, between vertices with n≡1,3 corresponding to the underlying Miura-ori strip, inserting vertices at n≡0,2). In particular, Fig. 3*F* is obtained from the Miura-ori strip in Fig. 3*B*, and exhibits a similar folding sequence due to the preservation of the mountain-valley assignment at the original Miura-ori vertices, which is also associated with a high packaging ratio. In summary, periodic degree-4 strips generically exhibit sequential deployment under adjacent-crease connections, with the transition width—and hence the number of involved unit cells—controlled by the effective parameter A/B of the composite map *f*.

### Programmable Macroscopic Shape While Preserving Propagation Behavior.

**Fig. 4.**
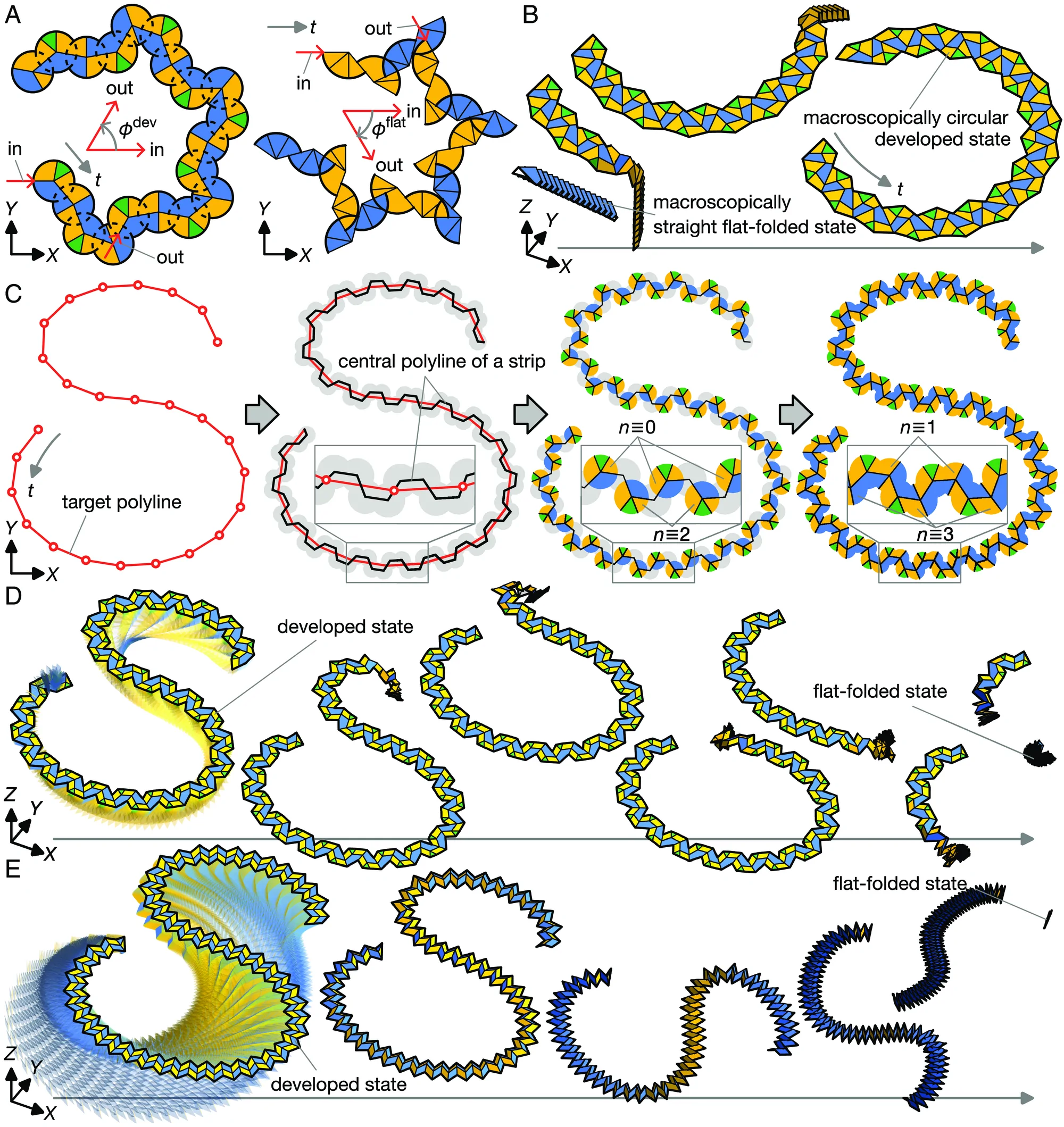
(*A*) Illustration of the total turning angle over one unit cell (N=4) in the developed state (*Left*) and the flat-folded state (*Right*). (*B*) Domino-like deployment of the periodic strip from the straight flat-folded state to the circular developed state [$N=4,{\left(\left(\theta_{n}^{0},\theta_{n}^{1}\right),i_{n}^{\text{out}},\sigma_{n}\right)}_{n=0,1,2,3}=\left(\left(\left(110^{{}^{\circ}},70^{{}^{\circ}}\right),1,-1\right),\left(\left(60^{{}^{\circ}},60^{{}^{\circ}}\right),2,1\right),\left(\left(110^{{}^{\circ}},70^{{}^{\circ}}\right),3,-1\right),\left(\left(130^{{}^{\circ}},130^{{}^{\circ}}\right),2,1\right)\right)$]. (*C*) Design procedure for a nonperiodic strip that deploys to a prescribed target shape. The period, output crease indices, mode assignment, and discrete dynamical system are identical to those in Fig. 3*F*. Given a target deployed shape represented as a polyline, each segment is first mapped to the central polyline of a unit cell. The sector angles of the vertices with n≡0,2 are then determined from $\theta_{0}^{0}$ and $\theta_{2}^{1}$, which are fixed by the central polyline, together with the prescribed effective parameter A/B. The sector angles of the vertices with n≡1,3 are subsequently determined. Although each vertex admits one degree of freedom in its sector angles, mirror-symmetric ones are selected (*D*). (*E*) Folding motions of strips that deploy into an S-shape via domino-like (*Top*) and uniform (*Bottom*) deployment. To compare swept volumes, snapshots of the folding motion are overlaid on the developed configurations.

The developed and flat-folded states of the examples in Fig. 3 *E* and *F* are both macroscopically straight when viewed along the central polyline connecting the vertices. More generally, for periodic strips, the macroscopic shapes of the developed and flat-folded states are characterized by the total turning angle over one unit cell (Fig. 4*A*),

[3] $$\begin{array}{c} \begin{array}{cc} \phi^{\text{dev}} & :=\sum_{n=0}^{N-1}\left(\pi +\sum_{i=1}^{i_{n}^{\text{out}}}\theta_{n}^{i-1}\right)\;\mathrm{mod}\;2\pi ,\\ \phi^{\text{flat}} & :=\sum_{n=0}^{N-1}\left(\pi +\sum_{i=1}^{i_{n}^{\text{out}}}{\left(-1\right)}^{s(n,i)}\theta_{n}^{i-1}\right)\mathrm{mod}\;2\pi ,\\ \text{where}\; & s(n,i):=i+\sum_{m=0}^{n-1}\left(i_{m}^{\text{out}}-1\right)\;\mathrm{mod}\;2,\\ & \phi^{\text{dev}},\phi^{\text{flat}}\in \left(-\pi ,\pi \right]. \end{array} \end{array}$$

The exponent s(n,i)≡0 (s(n,i)≡1) indicates that the *i*-th face of the *n*-th vertex is face-up (face-down) at the flat-folded state. The quantities $\phi^{\text{dev}}$ and $\phi^{\text{flat}}$ depend only on the output crease indices and sector angles and are independent of the crease lengths between the vertices, i.e., they represent a discrete curvature accumulated over one unit cell. For the examples in Fig. 3, these quantities evaluate to: $\phi^{\text{dev}}=-22.5^{{}^{\circ}}$ for Fig. 3*A* and 0 for Fig. 3*B*–*F*, while $\phi^{\text{flat}}=0$ for Fig. 3*B*, *E*, and *F*, and $22.5^{{}^{\circ}}$, $120^{{}^{\circ}}$, and $60^{{}^{\circ}}$ for Fig. 3*A*, *C*, and *D*, respectively. Building on these examples, the flat-folded and developed shapes can be controlled by tuning $\phi^{\text{dev}}$ and $\phi^{\text{flat}}$, which are in turn tuned by the sector angles. For example, the strip in Fig. 4*B*, derived from Fig. 3*F* by modifying the sector angles, deploys from a straight flat-folded state ($\phi^{\text{flat}}=0^{{}^{\circ}}$) into a circular developed state ($\phi^{\text{dev}}=20^{{}^{\circ}}$). However, for periodic strips, the total turning angle is constant across cells, leading only to straight or circular configurations. To realize more general shapes, spatial variations in the sector angles must be introduced. Such variations, however, typically render the recurrence relation nonautonomous—i.e., dependent on the cell index *t*—and thus lead to spatially varying propagation behavior. Here, as shown in Eq. **1**, the relation between opposite fold angles reduces to the identity; therefore, variations in the sector angles at vertices with $i_{n}^{\text{out}}=2$—for example, the n≡1,3 vertices in Fig. 3 *E* and *F*—do not affect the discrete dynamical system *f*. Furthermore, even for vertices with $i_{n}^{\text{out}}=1$ or 3, the recurrence relation can be preserved under variations of the sector angles that keep the effective parameter A/B invariant. These properties allow nonperiodic variations in the sector angles while keeping *f* autonomous, by ensuring that the recurrence relation is maintained at each vertex. Leveraging this principle, we construct an inverse-design algorithm based on the periodic strip in Fig. 3*F*, which determines the sector angles so that, while preserving the autonomous dynamical system specified by the effective parameter A/B, the strip develops into a configuration that follows a prescribed planar polyline (Fig. 4*C*; see *SI Appendix*, section 4 and Fig. S1 for computational details). Fig. 4*D* shows a strip designed using this algorithm that deploys sequentially into an S-shaped target configuration (Movie S9 and *SI Appendix*, Table S1 for the target polyline coordinates). For comparison, Fig. 4*E* shows a strip that deploys uniformly to the same target shape (Movie S10). By comparing the folding motions, we observe that the uniformly deploying strip (Fig. 4*E*) sweeps regions that deviate significantly from the target shape during deployment, whereas the present design (Fig. 4*D*) remains largely confined near the target configuration. To quantify this deviation, we evaluate the one-sided Hausdorff distance from the projected swept region of the central polyline during deployment (set $U\subset R^{2}$) to the target polyline (set $V\subset R^{2}$), defined as $\max_{u\in U}\min_{v\in V}\left\|v-u\right\|$, using a point-sampled approximation (*SI Appendix*, Fig. S2). The one-sided Hausdorff distance for Fig. 4*D* is approximately 0.37 times that for Fig. 4*E*. Moreover, the method in Fig. 4*C* extends to complex, maze-like targets, as illustrated by deployment along a Hilbert-curve-like polyline (Movie S11). These results demonstrate that the deployed shape of domino-deploying origami strips can be programmed independently of their propagation behavior. In addition, compared to uniformly deploying designs, the present approach keeps the swept region significantly closer to the target configuration, suggesting potential applications in deployment within confined or complex environments.

### Demonstration of the Domino-Like Deployment.

**Fig. 5.**
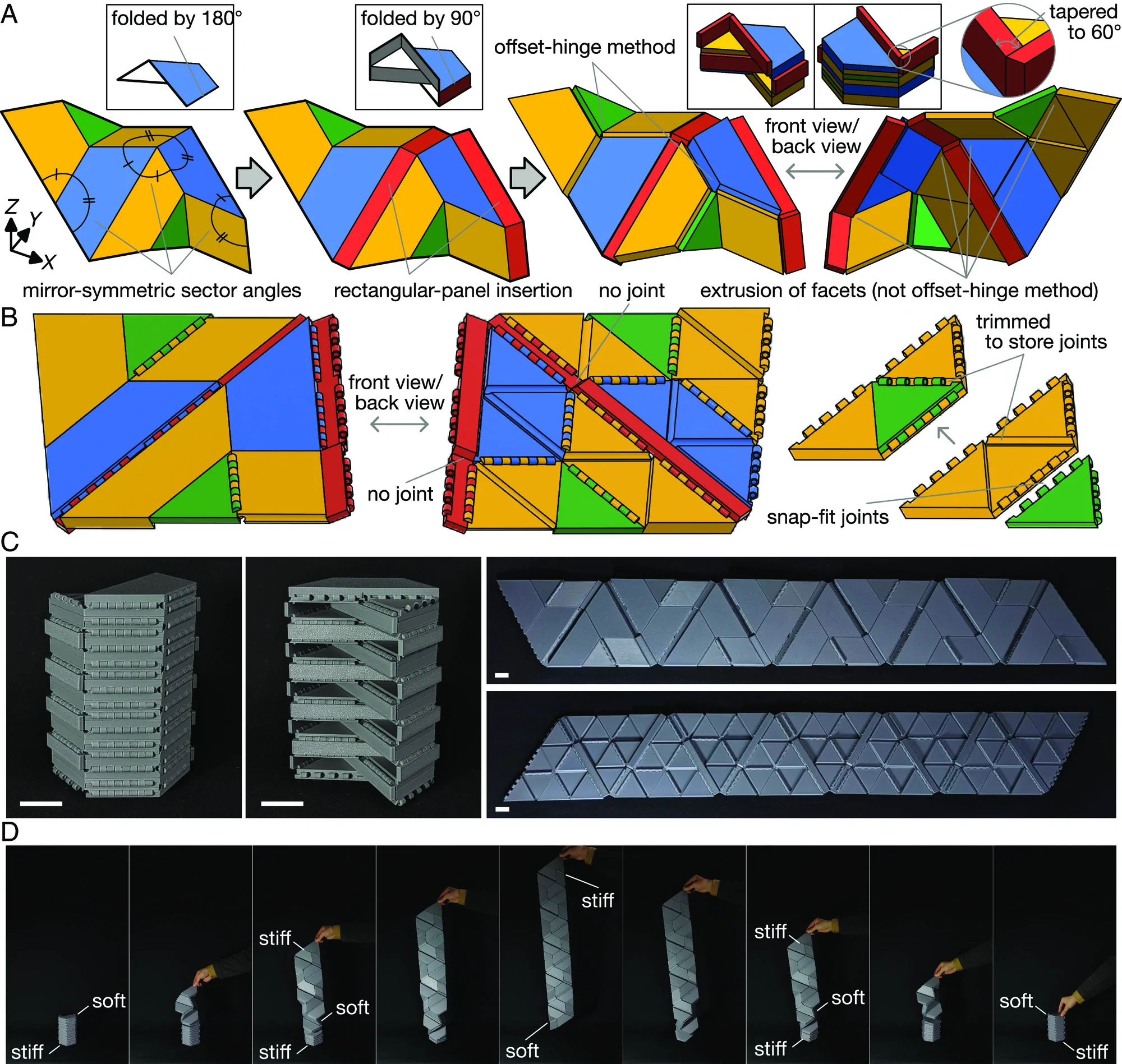
(*A*) Transformation process of a partially folded zero-thickness unit cell via rectangular-panel insertion and panel thickening using the face-extrusion and offset-hinge methods. *Insets* show the flat-folded states at each step. (*B*) Unit cell assembled from panels with snap-fit hinges placed along the creases. For simplicity, joints at the short creases between rectangular panels are omitted, as they do not affect the kinematics. Some panel corners are trimmed to accommodate the joints in the flat-folded state. (*C*) Prototype of the strip consisting of five unit cells (*Left*: *Front* and *Bottom* view of the flat-folded state; *Right*: *Front* and *Bottom* view of the developed state). (Scale bar, 2 cm.) Each panel was 3D printed using a Bambu Lab P1S with PLA filament. (*D*) Snapshots of the deployment and stowage of the prototype. The localized soft region corresponding to the transition between the flat-folded and developed states is initially located near the *Top* of the strip in the flat-folded configuration and gradually shifts toward the *Bottom* as deployment proceeds.

To demonstrate the feasibility of domino-like deployment, we constructed a physical prototype based on the design shown in Fig. 3*F*. To this end, we transformed the zero-thickness origami mechanism into a structure composed of panels with finite thickness while preserving its kinematics. Accounting for panel thickness prevents unintended elastic deformation of facets that may interfere with the kinematic coupling between vertices—an effect beyond the scope of this study—and is also essential for practical engineering applications such as deployable structures and robotics. Here, we employ the offset-hinge method, which is applicable to developable and flat-foldable degree-4 vertices (46–49). In this approach, each panel is assigned a thickness determined by the sector angles $\left(\theta^{0},\theta^{1}\right)$, and hinges are placed on the bottom (top) surfaces for mountain (valley) folds. In general, even if no self-intersection occurs in the zero-thickness origami, additional self-intersections may arise when it is transformed into a structure with finite-thickness panels, depending on the crease pattern and the thickening method. Indeed, when the offset-hinge method is applied to each vertex of the strip in Fig. 3*F*, panel self-intersection can occur; avoiding this requires the panel thickness to vary exponentially along the strip, rendering the design impractical (see *SI Appendix*, section 5 and Fig. S3 for details). To resolve this issue, we introduce rectangular panels at two of the four vertices (n≡1,3) in each unit cell (Fig. 5*A*). At each such vertex, the insertion of a rectangular panel can be represented by extruding creases i=1,3 along the normal to the plane spanned by them; hence, the input–output relation between fold angles is preserved. Furthermore, owing to the mirror symmetry of the sector angles at these vertices, the creases i=1,3 are effectively replaced by a pair of parallel creases, with the original fold angle equally distributed between them; in particular, these creases are folded at ±π/2 in the flat-folded state. This feature allows these vertices to be thickened without relying on the offset-hinge method; instead, the facets can be simply extruded along their normals. Consequently, the offset-hinge method needs to be applied only to the remaining two vertices (n≡0,2). This strategy yields a design with uniform panel thickness along the strip, although the deployed configuration becomes staircase-like (Movie S12). Finally, snap-fit hinges are incorporated into each panel, and local trimming is applied where necessary to prevent interference during folding (Fig. 5*B*). The panels are fabricated by 3D printing and assembled into a prototype (Fig. 5*C*; geometric dimensions of the corresponding zero-thickness and thick-panel origami are given in *SI Appendix*, Fig. S4). The structure exhibits the expected behavior: When deployed from one end, the transition propagates sequentially along the strip, reaching the fully deployed state (Fig. 5*D* and Movie S13). Conversely, during folding, the structure returns to the flat-folded state in the reverse order of deployment. Importantly, the opposite end remains mechanically stiff at the developed state, making it difficult to initiate folding from that side, which is analogous to topological polarization in mechanical systems (16, 18–20). This localization of the soft region is the mechanical manifestation of the kinematically programmed folding sequence. The localized soft region moves along the strip as the sequential deployment or stowage proceeds, corresponding to the transition region of a heteroclinic orbit or a sigmoid function. Regarding folding-mode selection, in zero-thickness origami, multiple folding branches can coexist at the developed state, requiring an explicit assignment of the folding mode, which is specified by *σ* in our parameterization. In the present thick-panel implementation, the offset-hinge design uniquely prescribes the mountain-valley assignment of the corresponding creases. On the other hand, the vertices thickened by rectangular-panel insertion and extrusion do not fully constrain the mountain-valley assignment, leaving a residual folding mode at the developed state; specifically, this residual mode allows the collinear creases of the rectangular panels to fold in the same direction. In the prototype, the mode assignment of the soft end vertex in the developed configuration is fixed by the offset-hinge geometry. We observed that, even from the developed configuration where the residual mode exists, actuating a single crease at the soft end vertex in the intended direction uniquely determines the mode assignment for the entire strip as prescribed. While the above discussion focuses on a specific prototype, the underlying thickness-accommodation method extends to other patterns. In particular, mirror symmetry of the sector angles at vertices with n≡1,3 is a necessary and sufficient condition for inserting rectangular panels without altering the kinematics. Under this condition, the strips in Fig. 4 *B* and *D* can be thickened in the same manner. In practice, however, the required panel trimming and hinge placement may become more intricate depending on the geometry. Despite these practical complexities, the qualitative agreement between the designed kinematics and the observed behavior demonstrates that domino-like deployment can be realized in physical origami structures.

## Discussion

In this work, we established a design framework for kinematic propagation of transition fronts in geometrically constrained systems, based on their correspondence with heteroclinic orbits in discrete dynamical systems. Using strips of developable, flat-foldable degree-4 origami vertices, we showed that asymmetric kinematic coupling between adjacent creases gives rise to domino-like sequential deployment without energy barriers, and that the developed shape can be programmed independently of the propagation behavior. In addition, the localized nature of the propagation suggests that such mechanisms may be advantageous for deployment under spatial constraints, where minimizing swept volume is critical. We further qualitatively validated this concept through a thick-panel origami prototype exhibiting the designed deployment.

Relaxing the developability condition may enable deployment from compact, flat-folded configurations to spatial shapes with nonzero torsion. Extending the proposed design framework to higher-dimensional structures, such as sheets and cellular assemblies, also remains an important direction for future work. Since the framework is formulated for serial mechanisms, such as the degree-4 origami strips considered here, it cannot be directly applied to sheet-like or cellular structures. Nevertheless, it can be extended to tree-like structures composed of multiple serial branches. Moreover, even for sheet-like or cellular structures, discrete dynamical system analysis may still be applicable if one-dimensional subsystems can be extracted (e.g., the Miura-ori strip shown in Fig. 3*B*) or if the structure can be reduced to a serial mechanism by imposing appropriate boundary conditions [e.g., tubular origami tessellations (36–38)]. Regardless of this analytical possibility, whether sequential deployment can be realized in sheet-like origami structures remains an open problem. Such extensions are expected to require additional design considerations, as many origami-based mesh or cellular structures (e.g., Miura-ori) are overconstrained and tend to exhibit uniform rather than domino-like deployment.

Importantly, because the propagation behavior is governed by an underlying discrete dynamical system, geometric parameters can be modified while preserving this structure. This enables design flexibility beyond programming the developed shape, such as simultaneous fitting of the developed and flat-folded configurations, tailoring point trajectories during deployment, minimizing swept volume, or tuning kinematic responses to specific functional requirements, without altering the propagation characteristics.

In the present study, we demonstrated the feasibility of domino-like deployment through qualitative observation of a representative physical prototype. Future work should include physical realization of a broader range of designs, together with systematic experimental and mechanical validation of the proposed framework, including assessments of practical deployability, robustness, and scalability. In particular, quan titative comparison between experimentally observed deploy ment behavior and the proposed kinematic theory, as well as quantitative characterization of the mechanical manifestations of the designed kinematic ordering, including the formation and propagation of stiff and soft regions during deployment, will be important for evaluating the applicability of the framework to practical engineering applications.

More broadly, this work establishes a kinematic foundation for understanding and designing transition fronts in geometrically constrained systems by linking local kinematic coupling to global propagation through discrete dynamical systems. By advancing from isolated examples toward a systematic design methodology, it opens possibilities for deployable structures, robotics, and mechanical metamaterials.

## Supplementary Material

Appendix 01 (PDF)

Movie S1. Visualization of the adjacent-fold-angle relation and the corresponding folding motion for several values of (*θ*^0^, *θ*^1^), arranged in a grid over the (*θ*^0^, *θ*^1^)-space. The sector angles are taken as *θ*^0^, *θ*^1^ ∈ {12.5^°^, 38.3333^°^, 64.1667^°^, 90^°^, 115.833^°^, 141.667^°^, 167.5^°^} with mode assignment σ = 1, excluding the singular cases where *θ*^1^ = π – *θ*^0^. The folding motion is generated by varying the initial fold angle *ρ*_0_ linearly at a constant rate. At each step, (*ρ*^0^, *ρ*^1^) and (*ρ*^0^, *ρ*^3^) corresponding to the folded state are plotted on the graph.

Movie S2. Visualization of the adjacent-fold-angle relation and the corresponding folding motion for several values of (*θ*^0^, *θ*^1^), arranged in a grid over the (*θ*^0^, *θ*^1^)-space. The sector angles are taken as *θ*^0^, *θ*^1^ ∈ {12.5^°^, 38.3333^°^, 64.1667^°^, 90^°^, 115.833^°^, 141.667^°^, 167.5^°^} with mode assignment σ = ‒1, excluding the singular cases where *θ*^1^ = *θ*^0^. The folding motion is generated by varying the initial fold angle *ρ*_0_ linearly at a constant rate. At each step, (*ρ*^0^, *ρ*^1^) corresponding to the folded state is plotted on the graph.

Movie S3. Folding motion of the strip shown in Fig. 3(A) of the main text. The motion is generated by varying the initial fold angle *ρ*_0_ according to *ρ*_0_(*t*_0_) = 2 arctan($p^{-t_{0}}$), where *t*_0_ ∈ [‒8, 24] and *p* ≈ 0.4875. The corresponding cobweb plot, orbit plot, and sigmoid function, along with its 10–90% transition width at each time step, are also shown.

Movie S4. Folding motion of the strip shown in Fig. 3(B) of the main text. The motion is generated by varying the initial fold angle *ρ*_0_ linearly at a constant rate. The evolution of the corresponding cobweb plot and phase-space trajectory at each time step is also shown.

Movie S5. Folding motion of the strip shown in Fig. 3(C) of the main text. The motion is generated by varying the initial fold angle *ρ*_0_ linearly at a constant rate. The evolution of the corresponding cobweb plot and phase-space trajectory at each time step is also shown.

Movie S6. Folding motion of the strip shown in Fig. 3(D) of the main text. The motion is generated by varying the initial fold angle *ρ*_0_ according to *ρ*_0_(*t*_0_) = ‒2 arctan($p^{-t_{0}}$), where *t*_0_ ∈ [‒7, 17] and *p* = 3. The corresponding cobweb plot, orbit plot, and sigmoid function, along with its 10–90% transition width at each time step, are also shown.

Movie S7. Folding motion of the strip shown in Fig. 3(E) of the main text. The motion is generated by varying the initial fold angle *ρ*_0_ according to *ρ*_0_(*t*_0_) = ‒2 arctan($p^{-t_{0}}$), where *t*_0_ ∈ [‒7, 13] and *p* = 4. The corresponding cobweb plot, orbit plot, and sigmoid function, along with its 10–90% transition width at each time step, are also shown.

Movie S8. Folding motion of the strip shown in Fig. 3(F) of the main text. The motion is generated by varying the initial fold angle *ρ*_0_ according to *ρ*_0_(*t*_0_) = ‒2 arctan($p^{-t_{0}}$), where *t*_0_ ∈ [‒7, 13] and *p* = 4. The corresponding cobweb plot, orbit plot, and sigmoid function, along with its 10–90% transition width at each time step, are also shown.

Movie S9. Folding motion of the strip shown in Fig. 4(D) of the main text. The motion is generated by varying the initial fold angle *ρ*_0_ according to *ρ*_0_(*t*_0_) = ‒2 arctan($p^{-t_{0}}$), where *t*_0_ ∈ [‒7, 32] and *p* = 4.

Movie S10. Folding motion of the strip shown in Fig. 4(E) of the main text. The motion is generated by varying the initial fold angle *ρ*_0_ linearly at a constant rate.

Movie S11. Folding motion of a strip that deploys along a Hilbert-curve-like polyline within a confined maze-like environment. The right panel shows the top view of the folding motion together with the target polyline (red). The motion is generated by varying the initial fold angle *ρ*_0_ according to *ρ*_0_(*t*_0_) = ‒2 arctan($p^{-t_{0}}$), where *t*_0_ ∈ [‒7, 395] and *p* = 4.

Movie S12. Folding motion of the thickness accommodated strip using the method shown in Fig. 5(A) of the main text.

Movie S13. Domino-like sequential deployment of the 3D-printed physical prototype shown in Fig. 5(D) of the main text. The prototype is initially placed in a flat-folded state on a planar surface, with the soft edges that should deploy first positioned on the upper side. As the upper panel is lifted, the structure undergoes sequential, one-degree-of-freedom deployment under gravity, until reaching the fully deployed state. In the deployed state, the bottom crease corresponds to a soft edge that should be folded first during stowage. Stowage is triggered when the prototype is lowered and contacts the planar surface. The structure then returns 216 to the flat-folded state in the reverse order of deployment, recovering its original flat-folded state. The video shows multiple repetitions of this deployment–stowage cycle.

Movie S14. Demonstration of the stiffness and softness of the edge creases of the prototype in the deployed state.

## Data Availability

Mathematica Notebooks and Data are openly available at Zenodo (50). All other data are included in the article and/or supporting information.
