# Supplementary material for: Programming sequential deployment of origami via kinematic transition fronts
Source: Proc Natl Acad Sci U S A. 2026 Sep 9;123(37):e2615468123. doi: 10.1073/pnas.2615468123 (PMC13578791; doi:10.1073/pnas.2615468123)
Supplement: Supplementary file 1 [file pnas.2615468123.sapp.pdf]

Supporting Information for

Programming sequential deployment of origami via kinematic transition fronts

Rinki Imada and Tomohiro Tachi

Rinki Imada.
E-mail: imada.rinki@jaxa.jp

This PDF file includes:

Supporting text
Figs. S1 to S4
Table S1
Legends for Movies S1 to S14
SI References

Other supporting materials for this manuscript include the following:

Movies S1 to S14

Supporting Information Text

1. Derivation of the Fold-Angle Relations

Here, we derive the fold-angle relations for a degree-4 vertex (Eq. (1) in the main text) using spherical trigonometry (see, e.g., (1, 2) for alternative but equivalent formulations and derivations). As illustrated in Fig. 1(A) of the main text, by "cutting out" a degree-4 vertex with a unit sphere centered at the internal vertex O, a spherical quadrilateral $P^0P^1P^2P^3$ is obtained, where P^i denotes the endpoint of the i-th crease. The sector angles $(\theta^i)_{i=0,1,2,3}$ and fold angles $(\rho^i)_{i=0,1,2,3}$ of the degree-4 vertex correspond to the edge lengths and the signed exterior angles of this spherical quadrilateral, respectively. Based on this correspondence, we derive the relations for opposite fold angles and adjacent fold angles in sequence. While the main text focuses on developable and flat-foldable vertices, the derivation here is presented for general vertices.

Opposite-fold-angle relations. We derive the relation between the opposite fold angles ρ^0 and ρ^2. By applying the law of cosines to the spherical triangles $\triangle P^0P^3P^1$ and $\triangle P^2P^3P^1$ with respect to their shared edge and combining the resulting equations, we obtain a relation between $\cos\rho^0$ and $\cos\rho^2$:

$$\begin{aligned}\cos\theta^3\cos\theta^0-\sin\theta^3\sin\theta^0\cos\rho^0&=\cos\theta^1\cos\theta^2-\sin\theta^1\sin\theta^2\cos\rho^2\\ \cos\rho^2&=\frac{\sin\theta^3\sin\theta^0}{\sin\theta^1\sin\theta^2}\cos\rho^0+\frac{\cos\theta^1\cos\theta^2-\cos\theta^3\cos\theta^0}{\sin\theta^1\sin\theta^2}.\end{aligned}\qquad [1]$$

Here, by introducing $\sigma:=\mathrm{sgn}(\rho^0)\mathrm{sgn}(\rho^2)$, as defined in the main text, we obtain the relation between ρ^2 and ρ^0:

$$\rho^2=\sigma\arccos\left(\frac{\sin\theta^3\sin\theta^0}{\sin\theta^1\sin\theta^2}\cos\rho^0+\frac{\cos\theta^1\cos\theta^2-\cos\theta^3\cos\theta^0}{\sin\theta^1\sin\theta^2}\right).\qquad [2]$$

Finally, by substituting $\theta^2=\pi-\theta^0$ and $\theta^3=\pi-\theta^1$ into Eq. (1) and Eq. (2), we obtain the following relation for developable and flat-foldable vertices:

$$\cos\rho^2=\cos\rho^0\quad\text{and}\quad\rho^2=\sigma\rho^0.\qquad [3]$$

Note that ρ^1 and ρ^3 also form an opposite pair; hence, $\cos\rho^3=\cos\rho^1$ holds.

Adjacent-fold-angle relations. We derive the relations between adjacent fold angles ρ^0 and ρ^1, as well as ρ^0 and ρ^3. We first consider the relation between ρ^0 and ρ^1. Let ξ^1 and ζ^1 denote the signed dihedral angles between the planes defined by (O,P^3,P^1) and (O,P^1,P^2), and between the planes defined by (O,P^3,P^1) and (O,P^0,P^1), respectively. Here, each plane is oriented so that the three defining points appear in counterclockwise order. Then, since $\rho^1=\xi^1+\zeta^1-\pi$, the following equation holds:

$$\cos\rho^1=-\cos\xi^1\cos\zeta^1+\sin\xi^1\sin\zeta^1\quad\text{and}\quad\sin\rho^1=-\sin\xi^1\cos\zeta^1-\cos\xi^1\sin\zeta^1.\qquad [4]$$

Using the law of cosines and the law of sines applied to the spherical triangle $\triangle P^0P^3P^1$, $\cos\xi^1$ and $\sin\xi^1$ can be expressed as follows:

$$\cos\xi^1=\frac{\cos\theta^3-(\cos\theta^3\cos\theta^0-\sin\theta^3\sin\theta^0\cos\rho^0)\cos\theta^0}{\sqrt{1-(\cos\theta^3\cos\theta^0-\sin\theta^3\sin\theta^0\cos\rho^0)^2}\sin\theta^0}\quad\text{and}\quad\sin\xi^1=\frac{-\sin\theta^3\sin\rho^0}{\sqrt{1-(\cos\theta^3\cos\theta^0-\sin\theta^3\sin\theta^0\cos\rho^0)^2}}.\qquad [5]$$

Similarly, using the law of cosines and the law of sines applied to the spherical triangle $\triangle P^2P^3P^1$, $\cos\zeta^1$ and $\sin\zeta^1$ can be expressed as follows:

$$\cos\zeta^1=\frac{\cos\theta^2-(\cos\theta^1\cos\theta^2-\sin\theta^1\sin\theta^2\cos\rho^2)\cos\theta^1}{\sqrt{1-(\cos\theta^1\cos\theta^2-\sin\theta^1\sin\theta^2\cos\rho^2)^2}\sin\theta^1}\quad\text{and}\quad\sin\zeta^1=\frac{-\sin\theta^2\sin\rho^2}{\sqrt{1-(\cos\theta^1\cos\theta^2-\sin\theta^1\sin\theta^2\cos\rho^2)^2}}.\qquad [6]$$

Substituting Eq. (5) and Eq. (6) into Eq. (4), we obtain the following:

$$\begin{aligned}\cos\rho^1&=\frac{-\left(\left(\sin\theta^1\cos\theta^2+\cos\theta^1\sin\theta^2\cos\rho^2\right)\left(\cos\theta^3\sin\theta^0+\sin\theta^3\cos\theta^0\cos\rho^0\right)\right)+\sin\theta^2\sin\theta^3\sin\rho^0\sin\rho^2}{\sqrt{1-(\cos\theta^1\cos\theta^2-\sin\theta^1\sin\theta^2\cos\rho^2)^2}\sqrt{1-(\cos\theta^3\cos\theta^0-\sin\theta^3\sin\theta^0\cos\rho^0)^2}},\\ \sin\rho^1&=\frac{\left(\sin\theta^1\cos\theta^2+\cos\theta^1\sin\theta^2\cos\rho^2\right)\sin\theta^3\sin\rho^0+\left(\cos\theta^3\sin\theta^0+\sin\theta^3\cos\theta^0\cos\rho^0\right)\sin\theta^2\sin\rho^2}{\sqrt{1-(\cos\theta^1\cos\theta^2-\sin\theta^1\sin\theta^2\cos\rho^2)^2}\sqrt{1-(\cos\theta^3\cos\theta^0-\sin\theta^3\sin\theta^0\cos\rho^0)^2}}.\end{aligned}\qquad [7]$$

Finally, substituting $\theta^2=\pi-\theta^0$, $\theta^3=\pi-\theta^0$, and $\rho^2=\sigma\rho^0$ into Eq. (7), we get the following equations:

$$\cos\rho^1=\frac{(\cos\theta^0\cos\theta^1+\sigma)\cos\rho^0+\sin\theta^0\sin\theta^1}{\sin\theta^0\sin\theta^1\cos\rho^0+(\cos\theta^0\cos\theta^1+\sigma)}\quad\text{and}\quad\sin\rho^1=\frac{-(\sigma\cos\theta^0+\cos\theta^1)\sin\rho^0}{\sin\theta^0\sin\theta^1\cos\rho^0+(\cos\theta^0\cos\theta^1+\sigma)}.\qquad [8]$$

The sign of the fold angle ρ^1 is determined by the sign of $\sin\rho^1$. Since the denominator is non-negative (non-positive) when $\sigma=1$ ($\sigma=-1$), ρ^1 is given as follows:

$$\rho^1=-\mathrm{sgn}(\rho^0)\mathrm{sgn}(\cos\theta^0+\sigma\cos\theta^1)\arccos\left(\frac{(\cos\theta^0\cos\theta^1+\sigma)\cos\rho^0+\sin\theta^0\sin\theta^1}{\sin\theta^0\sin\theta^1\cos\rho^0+(\cos\theta^0\cos\theta^1+\sigma)}\right).\qquad [9]$$

Another adjacent fold-angle relation between ρ^0 and ρ^3 can be derived in a similar manner. Let ξ^3 and ζ^3 denote the signed dihedral angles between the planes defined by (O, P^3, P^1) and (O, P^2, P^3), and between the planes defined by (O, P^3, P^1) and (O, P^3, P^0), respectively. Then, since $\rho^3 = \xi^3 + \zeta^3 - \pi$, the following equation holds:

$$\cos\rho^3 = -\cos\xi^3\cos\zeta^3 + \sin\xi^3\sin\zeta^3 \quad \text{and} \quad \sin\rho^3 = -\sin\xi^3\cos\zeta^3 - \cos\xi^3\sin\zeta^3. \tag{10}$$

Using the law of cosines and the law of sines applied to the spherical triangle $\triangle P^0P^3P^1$, $\cos\xi^3$ and $\sin\xi^3$ can be expressed as follows:

$$\cos\xi^3 = \frac{\cos\theta^0 - (\cos\theta^3\cos\theta^0 - \sin\theta^3\sin\theta^0\cos\rho^0)\cos\theta^3}{\sqrt{1-(\cos\theta^3\cos\theta^0 - \sin\theta^3\sin\theta^0\cos\rho^0)^2}\sin\theta^3} \quad \text{and} \quad \sin\xi^3 = \frac{-\sin\theta^0\sin\rho^0}{\sqrt{1-(\cos\theta^3\cos\theta^0 - \sin\theta^3\sin\theta^0\cos\rho^0)^2}}. \tag{11}$$

Similarly, using the law of cosines and the law of sines applied to the spherical triangle $\triangle P^2P^3P^1$, $\cos\zeta^3$ and $\sin\zeta^3$ can be expressed as follows:

$$\cos\zeta^3 = \frac{\cos\theta^1 - (\cos\theta^1\cos\theta^2 - \sin\theta^1\sin\theta^2\cos\rho^2)\cos\theta^2}{\sqrt{1-(\cos\theta^1\cos\theta^2 - \sin\theta^1\sin\theta^2\cos\rho^2)^2}\sin\theta^2} \quad \text{and} \quad \sin\zeta^3 = \frac{-\sin\theta^1\sin\rho^2}{\sqrt{1-(\cos\theta^1\cos\theta^2 - \sin\theta^1\sin\theta^2\cos\rho^2)^2}}. \tag{12}$$

Substituting Eq. (11) and Eq. (12) into Eq. (10), we obtain the following:

$$\begin{aligned}
\cos\rho^3 &= \frac{-\left(\left(\cos\theta^1\sin\theta^2 + \sin\theta^1\cos\theta^2\cos\rho^2\right)\left(\sin\theta^3\cos\theta^0 + \cos\theta^3\sin\theta^0\cos\rho^0\right)\right) + \sin\theta^0\sin\theta^1\sin\rho^0\sin\rho^2}{\sqrt{1-(\cos\theta^1\cos\theta^2 - \sin\theta^1\sin\theta^2\cos\rho^2)^2}\sqrt{1-(\cos\theta^3\cos\theta^0 - \sin\theta^3\sin\theta^0\cos\rho^0)^2}},\\
\sin\rho^3 &= \frac{\left(\cos\theta^1\sin\theta^2 + \sin\theta^1\cos\theta^2\cos\rho^2\right)\sin\theta^0\sin\rho^0 + \left(\sin\theta^3\cos\theta^0 + \cos\theta^3\sin\theta^0\cos\rho^0\right)\sin\theta^1\sin\rho^2}{\sqrt{1-(\cos\theta^1\cos\theta^2 - \sin\theta^1\sin\theta^2\cos\rho^2)^2}\sqrt{1-(\cos\theta^3\cos\theta^0 - \sin\theta^3\sin\theta^0\cos\rho^0)^2}}.
\end{aligned} \tag{13}$$

Finally, substituting $\theta^2 = \pi - \theta^0$, $\theta^3 = \pi - \theta^0$, and $\rho^2 = \sigma\rho^0$ into Eq. (13), we get the following equations:

$$\cos\rho^3 = \frac{(\cos\theta^0\cos\theta^1 + \sigma)\cos\rho^0 + \sin\theta^0\sin\theta^1}{\sin\theta^0\sin\theta^1\cos\rho^0 + (\cos\theta^0\cos\theta^1 + \sigma)} = \cos\rho^1 \quad \text{and} \quad \sin\rho^3 = \frac{(\cos\theta^0 + \sigma\cos\theta^1)\sin\rho^0}{\sin\theta^0\sin\theta^1\cos\rho^0 + (\cos\theta^0\cos\theta^1 + \sigma)} = -\sigma\sin\rho^1. \tag{14}$$

From Eq. (14), $\rho^3 = -\sigma\rho^1$ holds.

2. Computation and Visualization of Folded Configurations

We describe how the folded configurations of the strips shown in Figs. 3 and 4 in the main text are visualized in three-dimensional space. The folded state of a strip is computed recursively, in a manner analogous to the fold angles, by sequentially determining the configuration of each degree-4 vertex. Although the strips considered in the main text are developable and flat-foldable, the representation and computational procedure described below are applicable more generally to vertices and strips that do not necessarily satisfy these conditions. Accordingly, we first describe the representation and computation of the folded state of a single degree-4 vertex, followed by those for the entire strip.

Vertex configuration. The folded state of a degree-4 vertex with sector angles $(\theta^i)_{i=0,1,2,3}$ can be determined by specifying the fold angle ρ^0 and computing the remaining fold angles $(\rho^i)_{i=1,2,3}$ according to Eq. (1) in the main text. However, to specify its spatial configuration in three-dimensional space, both position and orientation must be defined. Here, we represent the position and orientation using the following quantities: the position of the central vertex $\mathbf{o} \in \mathbb{R}^3$, unit vectors $\mathbf{c}^i \in \mathbb{R}^3$ indicating the directions of the creases, and unit normal vectors $\mathbf{n}^i \in \mathbb{R}^3$ of the faces. Each vector $\mathbf{c}^i$ is based at $\mathbf{p}$. The vector $\mathbf{n}^i$ denotes the unit normal of the face bounded by the i-th and $(i+1)$-th creases, and the configuration of this face is represented by the triplet $(\mathbf{o}, \mathbf{c}^i, \mathbf{n}^i)$. Once the folded state is specified, i.e., when $(\rho^i)_{i=0,1,2,3}$ are given, the configurations of the four faces can be computed recursively from the configuration of the 0-th face, $(\mathbf{o}, \mathbf{c}^0, \mathbf{n}^0)$, using the relations

$$\mathbf{c}^{i+1} = \mathbf{R}(\theta^i, \mathbf{n}^i)\mathbf{c}^i \quad \text{and} \quad \mathbf{n}^{i+1} = \mathbf{R}(\rho^{i+1}, \mathbf{c}^{i+1})\mathbf{n}^i, \tag{15}$$

where $\mathbf{R}(\theta, \mathbf{v}) \in \mathbb{R}^{3\times 3}$ denotes the rotation matrix representing a rotation by angle θ about the axis $\mathbf{v}$. From the above, the folded state and spatial configuration of a degree-4 vertex can be fully specified by ρ^0 together with $(\mathbf{o}, \mathbf{c}^0, \mathbf{n}^0)$.

Strip configuration. Next, we consider the configuration of a strip specified by the sequence of sector angles and output-crease indices, $((\theta_n^i)_{i=0,1,2,3}, i_n^{\mathrm{out}})_{n=0,1,\ldots}$. We also assign the crease lengths at the n-th vertex as $(l_n^i)_{i=0,1,2,3}$ where $l_n^i \in \mathbb{R}_{>0}$; from the connectivity between adjacent vertices, these satisfy $l_{n+1}^0 = l_n^{i_0^{\mathrm{out}}}$. The folded state of the strip is determined by specifying the input fold angle ρ_0^0 at the 0-th vertex and computing all other fold angles using Eq. (1) in the main text. Following the single-vertex formulation, we represent the configuration of the n-th vertex by $\mathbf{o}_n$, $(\mathbf{c}_n^i)_{i=0,1,2,3}$, and $(\mathbf{n}_n^i)_{i=0,1,2,3}$, and use these sequences to describe the configuration of the entire strip. The configuration of the 0-th vertex can be computed from Eq. (15) once $\mathbf{c}_0^0$ and $\mathbf{n}_0^0$ are specified. From the connectivity between the 0-th and 1-st vertices, we have

$$\mathbf{o}_1 = \mathbf{o}_0 + l_0^{i_0^{\mathrm{out}}}\mathbf{c}_0^{i_0^{\mathrm{out}}}, \quad \text{and} \quad \mathbf{c}_1^0 = -\mathbf{c}_0^{i_0^{\mathrm{out}}}. \tag{16}$$

Furthermore, since the 0-th face of the 1-st vertex lies in the same plane as the $(i_0^{\text{out}}-1)$-th face of the 0-th vertex, we have

$$\mathbf{n}_1^0 = \mathbf{n}_0^{i_0^{\text{out}}-1}. \tag{17}$$

Thus, the configuration of the 1-st vertex can be computed from Eq. (15). Generalizing this procedure, the parameters representing the configuration of the $(n+1)$-th vertex are determined from those of the n-th vertex as

$$\mathbf{o}_{n+1} = \mathbf{o}_n + l_n^{i_n^{\text{out}}} \mathbf{c}_n^{i_n^{\text{out}}}, \quad \mathbf{c}_{n+1}^0 = -\mathbf{c}_n^{i_n^{\text{out}}}, \quad \text{and} \quad \mathbf{n}_{n+1}^0 = \mathbf{n}_n^{i_n^{\text{out}}-1}. \tag{18}$$

By this recursive procedure, the folded state and spatial configuration of the strip can be fully determined from ρ_0^0 and $(\mathbf{o}_0, \mathbf{c}_0^0, \mathbf{n}_0^0)$, using Eq. (15) and Eq. (18). In Figs. 3 and 4 of the main text, each strip is visualized by placing points along the lateral creases according to the crease lengths $(l_n^i)_{i=0,1,2,3}$, and constructing faces by connecting sets of coplanar points.

3. Derivation of the Sigmoid Function

We derive the sigmoid function defined by Eq. (2) in the main text. As stated in the main text, $\cos\rho^1$ and $\cos\rho^0$ are related by the following symmetric linear fractional transformation:

$$\cos\rho^1 = \frac{A\cos\rho^0 + B}{B\cos\rho^0 + A}, \quad \text{where} \quad A := \cos\theta^0\cos\theta^1 + \sigma, \quad B := \sin\theta^0\sin\theta^1. \tag{19}$$

Applying the tangent half-angle substitution to Eq. (19), we obtain the following:

$$\begin{aligned} \frac{1-\tan^2\dfrac{\rho^1}{2}}{1+\tan^2\dfrac{\rho^1}{2}} &= \frac{A\dfrac{1-\tan^2\dfrac{\rho^0}{2}}{1+\tan^2\dfrac{\rho^0}{2}} + B}{B\dfrac{1-\tan^2\dfrac{\rho^0}{2}}{1+\tan^2\dfrac{\rho^0}{2}} + A} \\ \tan^2\frac{\rho^1}{2} &= \frac{A-B}{A+B}\tan^2\frac{\rho^0}{2}. \end{aligned} \tag{20}$$

Therefore, taking the sign into account, ρ^1 is given as follows:

$$\tan\frac{\rho^1}{2} = -\operatorname{sgn}(\cos\theta^0 + \sigma\cos\theta^1)\sqrt{\frac{A-B}{A+B}}\tan\frac{\rho^0}{2}. \tag{21}$$

Therefore, in the example shown in Fig. 3(A) considered in Eq. (2) of the main text, the general term ρ_t of the input fold-angle sequence $(\rho_t)_{t=0,\dots}$ can be expressed as follows:

$$\rho_t = 2\arctan\left(\left(\tan\frac{\rho^0}{2}\right)p^t\right), \quad \text{where} \quad p := -\operatorname{sgn}(\cos\theta^0 + \sigma\cos\theta^1)\sqrt{\frac{A-B}{A+B}}\tan\frac{\rho^0}{2}. \tag{22}$$

If the index t in Eq. (22) expression is formally extended to take real values, it yields the sigmoid function described in the main text. As also noted in the main text, since symmetric linear fractional transformations are closed under composition, sigmoid functions corresponding to examples other than Fig. 3(A) can be obtained by replacing A and B with the parameters of the composed transformation.

4. Mapping a Target Polyline to the Crease Pattern of the Strip

We describe how the input polyline $(\mathbf{p}_t)_{t=0,\dots,T}$ with $\mathbf{p}_t \in \mathbb{R}^2$ is transformed into the central crease $(\mathbf{o}_n)_{n=0,\dots,4T-1}$ with $\mathbf{o}_n \in \mathbb{R}^2$ in Fig. 4(C) of the main text. For simplicity, we assume that both the input polyline and the central crease have uniform segment lengths, denoted by $L \in \mathbb{R}_{>0}$ and $l \in \mathbb{R}_{>0}$, respectively. As a natural construction, the t-th segment of the polyline is associated with the central crease of one unit cell, represented by $(\mathbf{o}_{4t}, \mathbf{o}_{4t+1}, \mathbf{o}_{4t+2}, \mathbf{o}_{4t+3})$. We further impose, as a design requirement, that when a straight polyline is given as input, the developed configuration shown in Fig. 3(F) of the main text is reproduced (up to a suitable adjustment). As illustrated in Fig. S1(A), connecting the midpoints of the input creases of each unit cell in the developed configuration of Fig. 3(F) yields a straight polyline. Based on this observation, we assign the endpoints of each segment to the midpoints of the input and output creases of each unit cell. Under this construction, the mapping of the t-th segment to the central crease of the t-th cell can be parameterized by two quantities: (1) the turning angle ϕ_t from the unit direction vector of the t-th segment, $\mathbf{v}_t := (\mathbf{p}_{t+1} - \mathbf{p}_t)/\|\mathbf{p}_{t+1} - \mathbf{p}_t\|$, to the vector $(\mathbf{o}_{4t} - \mathbf{p}_t)/\|\mathbf{o}_{4t} - \mathbf{p}_t\|$, and

(2) the turning angle ψ_t from $(\mathbf{o}_{4t+3}-\mathbf{o}_{4t})/\|\mathbf{o}_{4t+3}-\mathbf{o}_{4t}\|$ to $(\mathbf{o}_{4t+1}-\mathbf{o}_{4t})/\|\mathbf{o}_{4t+1}-\mathbf{o}_{4t}\|$ (Fig. S1(B)). Using these parameters, the positions of the four vertex centers within the t-th unit cell in the developed state are given by

$$\begin{aligned}
\mathbf{o}_{4t} &= \mathbf{p}_t + \frac{l}{2}\mathbf{R}(\phi_t)\,\mathbf{v}_t,\\
\mathbf{o}_{4t+3} &= \mathbf{p}_{t+1} - \frac{l}{2}\mathbf{R}(\phi_{t+1})\,\mathbf{v}_{t+1},\\
\mathbf{o}_{4t+1} &= \mathbf{o}_{4t} + l\,\mathbf{R}(\psi_t)\left(\frac{\mathbf{o}_{4t+3}-\mathbf{o}_{4t}}{\|\mathbf{o}_{4t+3}-\mathbf{o}_{4t}\|}\right),\\
\mathbf{o}_{4t+2} &= \mathbf{o}_{4t+3} + l\,\mathbf{R}(\bar{\psi}_t)\left(\frac{\mathbf{o}_{4t}-\mathbf{o}_{4t+3}}{\|\mathbf{o}_{4t}-\mathbf{o}_{4t+3}\|}\right).
\end{aligned} \tag{23}$$

Here, $\mathbf{R}(\phi)\in\mathbb{R}^{2\times 2}$ denotes the two-dimensional rotation matrix with rotation angle ϕ, and $\bar{\psi}_t$ is defined as

$$\begin{aligned}
\bar{\psi}_t &:= \arccos\left(\left(\frac{a^2}{2la}\right)\left(\frac{a^2+\|\mathbf{o}_{4t+3}-\mathbf{o}_{4t}\|^2-l^2}{2a\|\mathbf{o}_{4t+3}-\mathbf{o}_{4t}\|}\right)+\sqrt{1-\left(\frac{a^2}{2la}\right)^2}\sqrt{1-\left(\frac{a^2+\|\mathbf{o}_{4t+3}-\mathbf{o}_{4t}\|^2-l^2}{2a\|\mathbf{o}_{4t+3}-\mathbf{o}_{4t}\|}\right)^2}\right),\\
&\text{where}\quad a := \sqrt{l^2+\|\mathbf{o}_{4t+3}-\mathbf{o}_{4t}\|^2-2l\|\mathbf{o}_{4t+3}-\mathbf{o}_{4t}\|\cos\psi_t}.
\end{aligned} \tag{24}$$

Instead of introducing and optimizing an explicit objective function, we determine ϕ_t and ψ_t heuristically. Specifically, ϕ_t is prescribed using the turning angle of the polyline segments as

$$\begin{aligned}
\phi_t &= \phi^* + \frac{\Delta\phi(\mathbf{v}_{t-1},\mathbf{v}_t)}{2},\\
\text{where}\quad \Delta\phi([x_1,y_1]^T,[x_2,y_2]^T) &:= \operatorname{atan2}\big(x_1y_2-y_1x_2,\ x_1x_2+y_1y_2\big).
\end{aligned} \tag{25}$$

Eq. (25) sets the target angle ϕ^* when the polyline is locally straight, and adjusts ϕ_t according to the deviation from straightness, measured by $\Delta\phi$. The initial rotation angle ϕ_0 at the starting point can be chosen arbitrarily. Next, ψ_t is prescribed by enforcing symmetry, i.e., $\psi_t=\bar{\psi}_t$, as

$$\psi_t = \arccos\left(\frac{L^2+(\|\mathbf{o}_{4t+3}-\mathbf{o}_{4t}\|/2)^2-(L/2)^2}{2L(\|\mathbf{o}_{4t+3}-\mathbf{o}_{4t}\|/2)}\right). \tag{26}$$

Eq. (25) and Eq. (26) provide a reasonable setting to reproduce the pattern shown in Fig. 3(F). Specifically, by inputting a straight polyline with segment length $L=1$ and substituting Eq. (25) and Eq. (26) into Eq. (23) under the parameters $l=1/3$ and $\phi^*=\phi_0=\pi/3$, we recover the pattern shown in Fig. 3(F) of the main text. The central creases shown in Fig. 4(C) and 4(D) of the main text are obtained by applying the above procedure to an S-shaped input polyline with segment length $L=1$, using the same parameters $l=1/3$ and $\phi^*=\phi_0=\pi/3$. After obtaining the central polyline of the strip, the next step is to calculate the sector angles at the vertices with $n\equiv 0,2$. For $n\equiv 0$, θ_n^0 is fixed by the central polyline. Given θ_n^0 and the value of $A/B=-5/3$, θ_n^1 is computed as

$$\theta_n^1 = \arccos\left(\frac{\cot\theta_n^0\csc\theta_n^0+\sqrt{\left(\frac{A}{B}\right)^2\left(\left(\frac{A}{B}\right)^2-1\right)}}{\left(\frac{A}{B}\right)^2+\cot^2\theta_n^0}\right). \tag{27}$$

Similarly, for $n\equiv 2$, using $\theta_n^1=\pi-\theta_n^3$ fixed by the central polyline and $A/B=-5/3$, θ_n^0 is computed as

$$\theta_n^0 = \arccos\left(\frac{\cot\theta_n^1\csc\theta_n^1-\sqrt{\left(\frac{A}{B}\right)^2\left(\left(\frac{A}{B}\right)^2-1\right)}}{\left(\frac{A}{B}\right)^2+\cot^2\theta_n^1}\right). \tag{28}$$

For vertices with $n\equiv 1,3$, the sector angles are determined so as to satisfy $\theta_n^0=\theta_n^1$. This procedure yields the crease patterns shown in Fig. 4(C) and (D). On the other hand, the central crease of the Miura-ori strip shown in Fig. 4(E) is constructed from the same input polyline, with $l=1/3$ and $\phi^*=\phi_0=40^\circ$, by reversing the signs of the rotation angles ψ_t and $\bar{\psi}_t$ in Eq. (23). The sector angles are then determined so that $\theta_n^0=\theta_n^1$ holds.

5. Thickness Accommodation of the Strip by the Offset-Hinge Method

Here, we first briefly introduce the offset-hinge method (3–6), a thickness-accommodation technique for developable and flat-foldable degree-4 vertices, focusing on its underlying principle and design conditions. We then show that, when the offset-hinge method is applied to each vertex of the strip shown in Fig. 3(F) in the main text, the panel thickness varies exponentially along the strip.

Introduction to offset-hinge method. Following previous work (4), we introduce the offset-hinge method. Fig. S3(A) and (B) show an example of applying the offset-hinge method to a degree-4 vertex. Focusing on the relative heights of the hinges in the developed state, the valley crease lies at the highest position, the mountain crease opposite to it lies at the lowest position, and the remaining mountain creases are located at intermediate heights. In the figure, the distances between adjacent creases, $(d^i)_{i=0,1,2,3}$, determine the thickness of the panels. Importantly, the values of $(d^i)_{i=0,1,2,3}$ are not arbitrary; to preserve kinematic equivalence, they must satisfy

$$d^1 = d^0 \frac{\sin\theta^1}{\sin\theta^0}, \quad d^2 = d^0, \quad \text{and} \quad d^3 = d^1. \qquad [29]$$

Eq. (29) arises from the correspondence between origami kinematics and linkage mechanisms: zero-thickness origami, in which creases intersect at a point, can be regarded as a 4R spherical linkage, whereas thickened origami, in which creases no longer intersect at a point, corresponds to a 4R spatial linkage. In this correspondence, the design parameters $(\theta^i)_{i=0,1,2,3}$ and $(d^i)_{i=0,1,2,3}$ correspond to the twist angles and link lengths of the 4R spatial linkage, respectively. Unlike a 4R spherical linkage, which is mobile for arbitrary sector angles, a 4R spatial linkage is generally overconstrained and exhibits mobility only when its twist angles and link lengths satisfy specific conditions. The developability and flat-foldability conditions of a single-vertex origami, together with Eq. (29), correspond to one such condition under which the 4R spatial linkage becomes an overconstrained but movable mechanism, known as the Bennett linkage. Therefore, when thickening a developable and flat-foldable degree-4 vertex using the offset-hinge method, the design has only one independent parameter, namely a single link length.

Exponential change of panel-thickness along the strip. When the offset-hinge method is applied to each vertex of the strip shown in Fig. 3(F) in the main text, the representative link lengths at each vertex, $(d_n^0)_{n=0,1,\dots}$, serve as the design parameters. In structures such as quadrilateral meshes, where internal vertices form loops, the link lengths must satisfy certain compatibility conditions in order to preserve mobility (5). In contrast, a strip contains no such loops; therefore, mobility is preserved for arbitrary choices of $(d_n^0)_{n=0,1,\dots}$. However, the situation changes when self-intersection between panels is taken into account. To avoid self-intersection in the flat-folded state, the developed configuration must be geometrically flat. As a result, once d_0^0 is specified, all other link lengths are uniquely determined. Consequently, the panel thickness is governed by the relative heights of the hinges in the deployed state. For the connectivity and mountain–valley assignment considered here, the relative height between the input and output creases remains unchanged at vertices with $n \equiv 1, 3$, where opposite creases connect adjacent vertices. In contrast, at vertices with $n \equiv 0, 2$, where adjacent creases are used for connectivity, the output crease is always located at a higher position than the input crease. As a result, the hinge positions increase monotonically with the cell index. The combination of these two effects leads to an exponential decrease in panel thickness along the strip as the cell index increases (Fig. S3(C)). The thickness-accommodation method presented in the main text avoids this exponential variation by employing extrusion, which eliminates the requirement that the deployed configuration be geometrically flat.

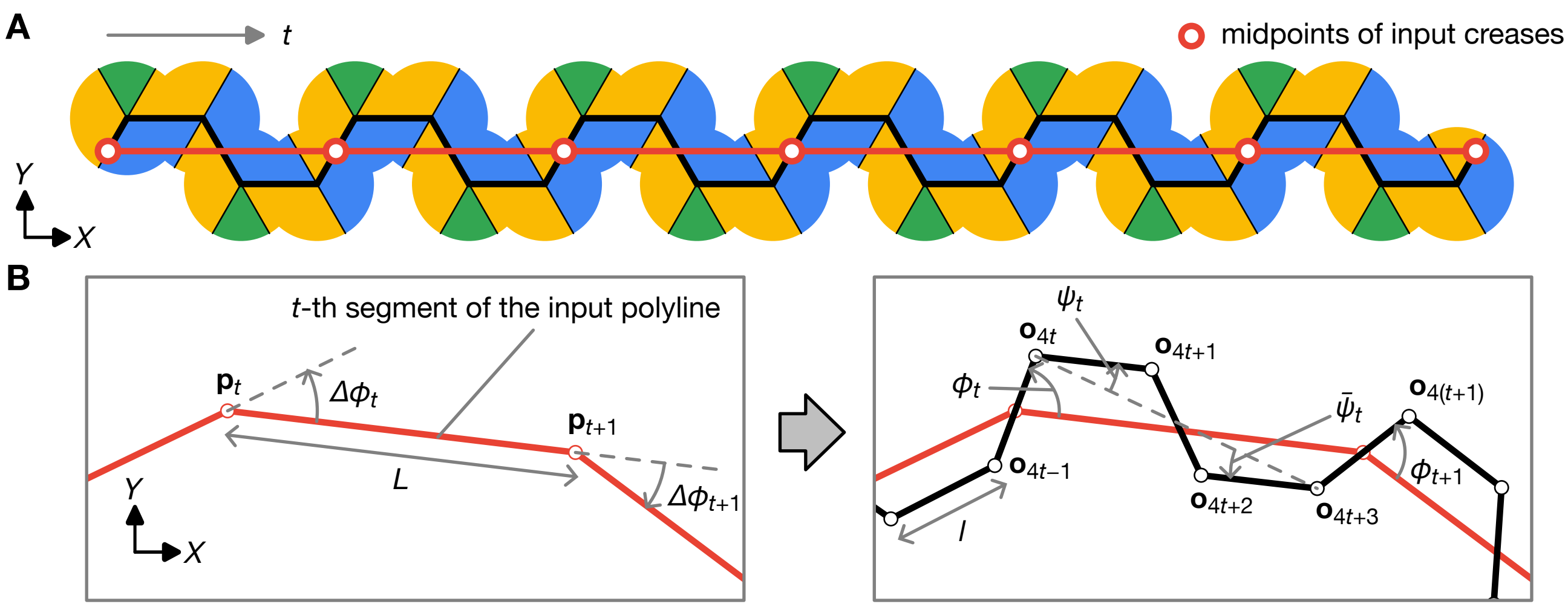

Fig. S1. (A) Straight polyline drawn on the crease pattern of the strip in Fig. 3(F) of the main text, connecting the midpoints of the input creases. (B) Mapping of the t-th segment of the input polyline (left) into the central creases of the t-th cell (right).

Table S1. Coordinates $(\mathbf{p}_t)_{t=0,\ldots,25}$ of the S-shaped target polyline used to generate the examples in Fig. 4(D) and (E) of the main text.

t	*x*	*y*
0	0.	0.
1	−0.612623	−0.790375
2	−0.751693	−1.78066
3	−0.333554	−2.68904
4	0.429971	−3.33482
5	1.35262	−3.72046
6	2.34043	−3.87613
7	3.33862	−3.81593
8	4.29816	−3.53437
9	5.1478	−3.00701
10	5.74809	−2.20722
11	5.86688	−1.2143
12	5.43235	−0.313643
13	4.66079	0.322515
14	3.73463	0.699646
15	2.74564	0.847633
16	1.74966	0.93724
17	0.817563	1.29944
18	0.129631	2.02521
19	0.169315	3.02442
20	0.892817	3.71475
21	1.83486	4.05024
22	2.83312	4.10922
23	3.81213	3.90541
24	4.6693	3.39037
25	5.0939	2.48499

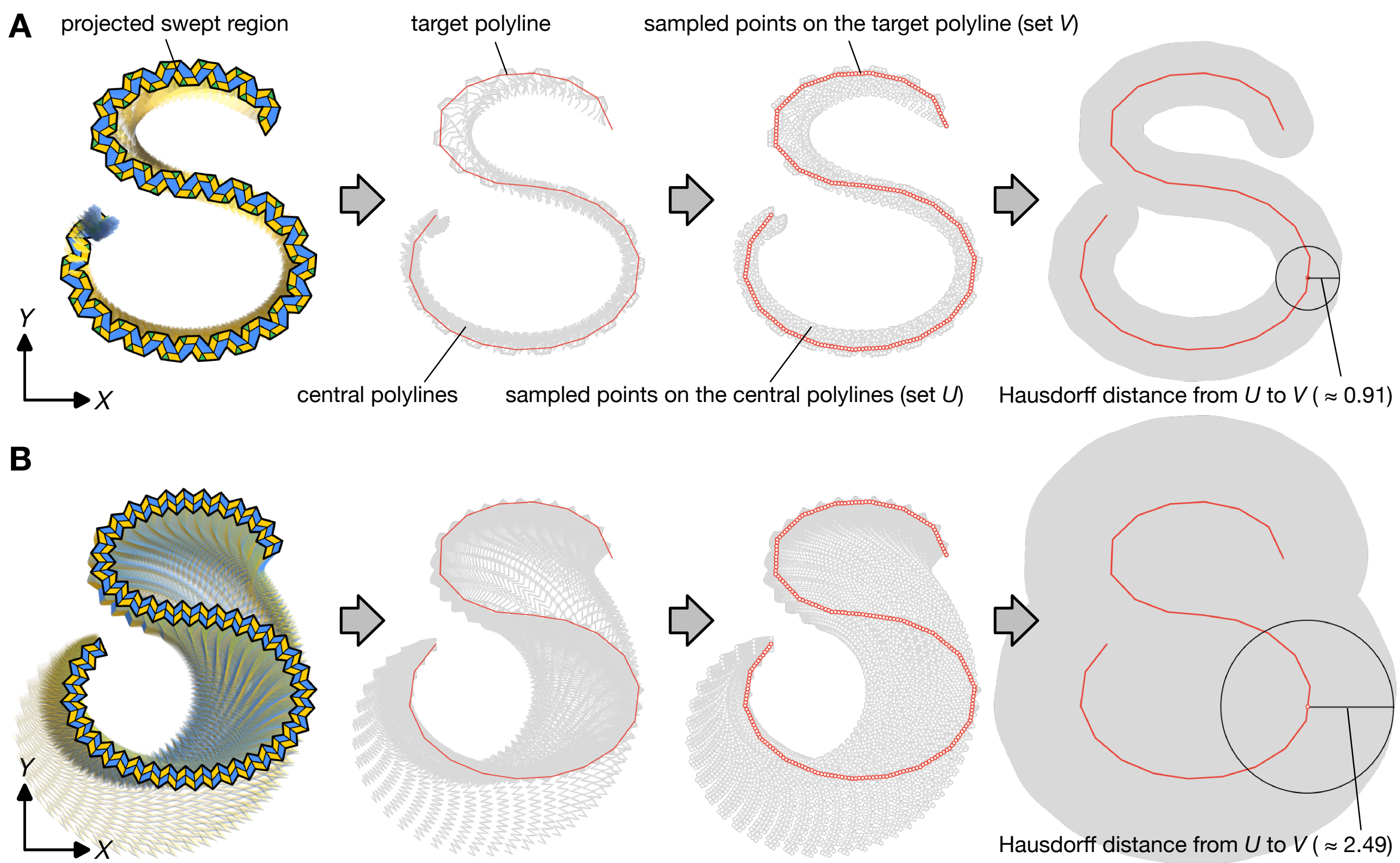

Fig. S2. Computation of the one-sided Hausdorff distance from the projected swept region of the central polyline during deployment (set $U \subset \mathbb{R}^2$) to the target polyline (set $V \subset \mathbb{R}^2$) using a point-sampled approximation. (A), (B) Procedure for computing the one-sided Hausdorff distance for Fig. 4(D) and (E) in the main text, respectively. First, we project the swept region of the strip onto the XY plane. Second, we extract the central polyline from each sampled folded state. Third, we sample points from the target polyline and from the family of the strip's central polylines to obtain the sets V and U, respectively. Finally, we calculate the one-sided Hausdorff distance from U to V, defined as $\max_{\mathbf{u}\in U} \min_{\mathbf{v}\in V} \|\mathbf{v} - \mathbf{u}\|$. The resulting values are 0.91 and 2.49 for Fig. 4(D) and (E), respectively. By definition, all points in U are contained in the union of disks centered at the points in V, each with radius equal to the computed one-sided Hausdorff distance (gray region in the right panel).

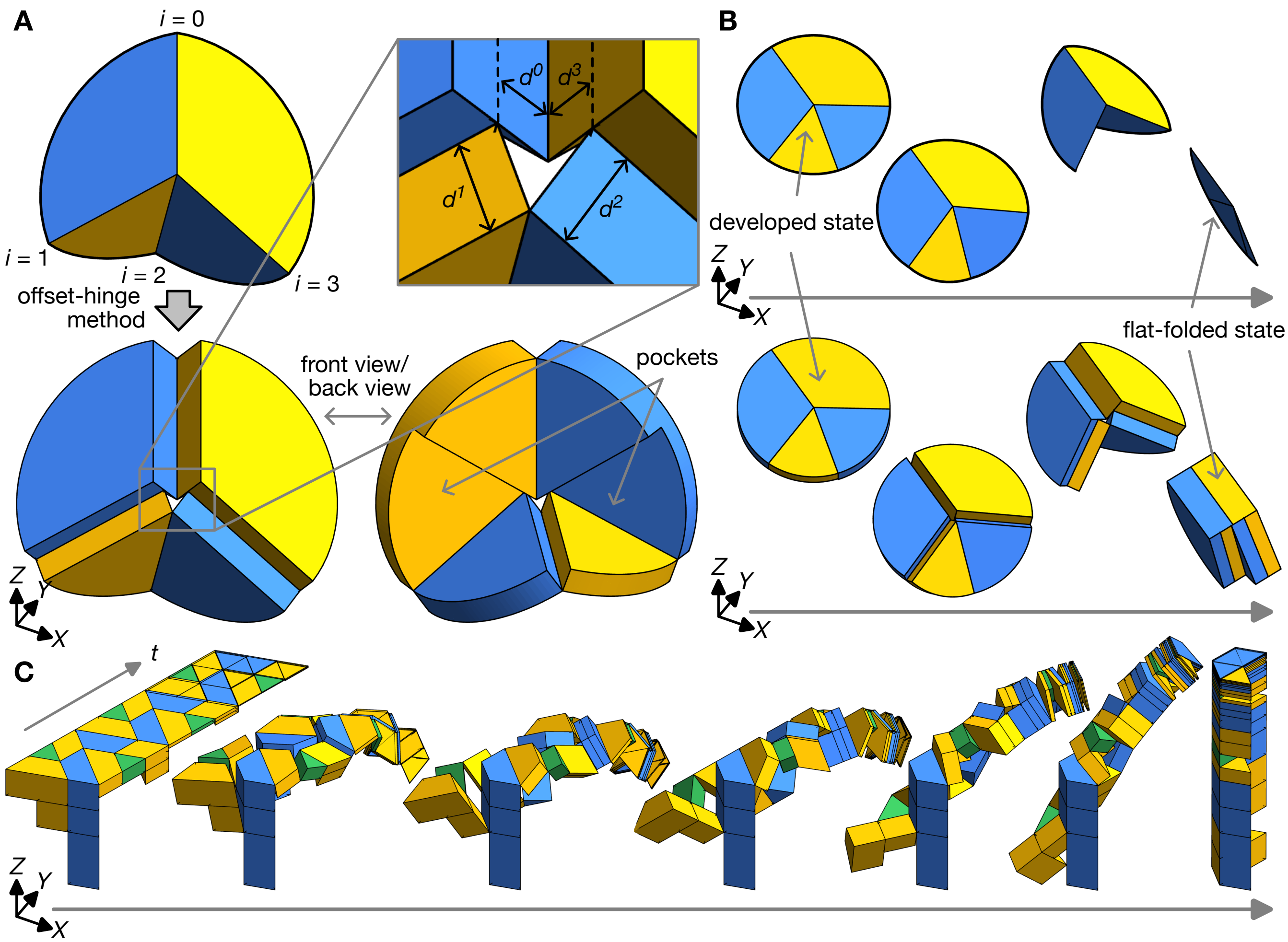

Fig. S3. (A) Thickening of a developable and flat-foldable degree-4 vertex using the offset-hinge method. The panel thickness is determined by the offset distances between adjacent creases, parameterized by $(d^i)_{i=0,1,2,3}$. (B) Equivalence of the folding motions before and after applying the offset-hinge method. (C) Snapshots of the folding motion of the strip exhibiting an exponential variation in panel thickness. Despite consisting of only three unit cells, the panel thickness varies significantly along the strip.

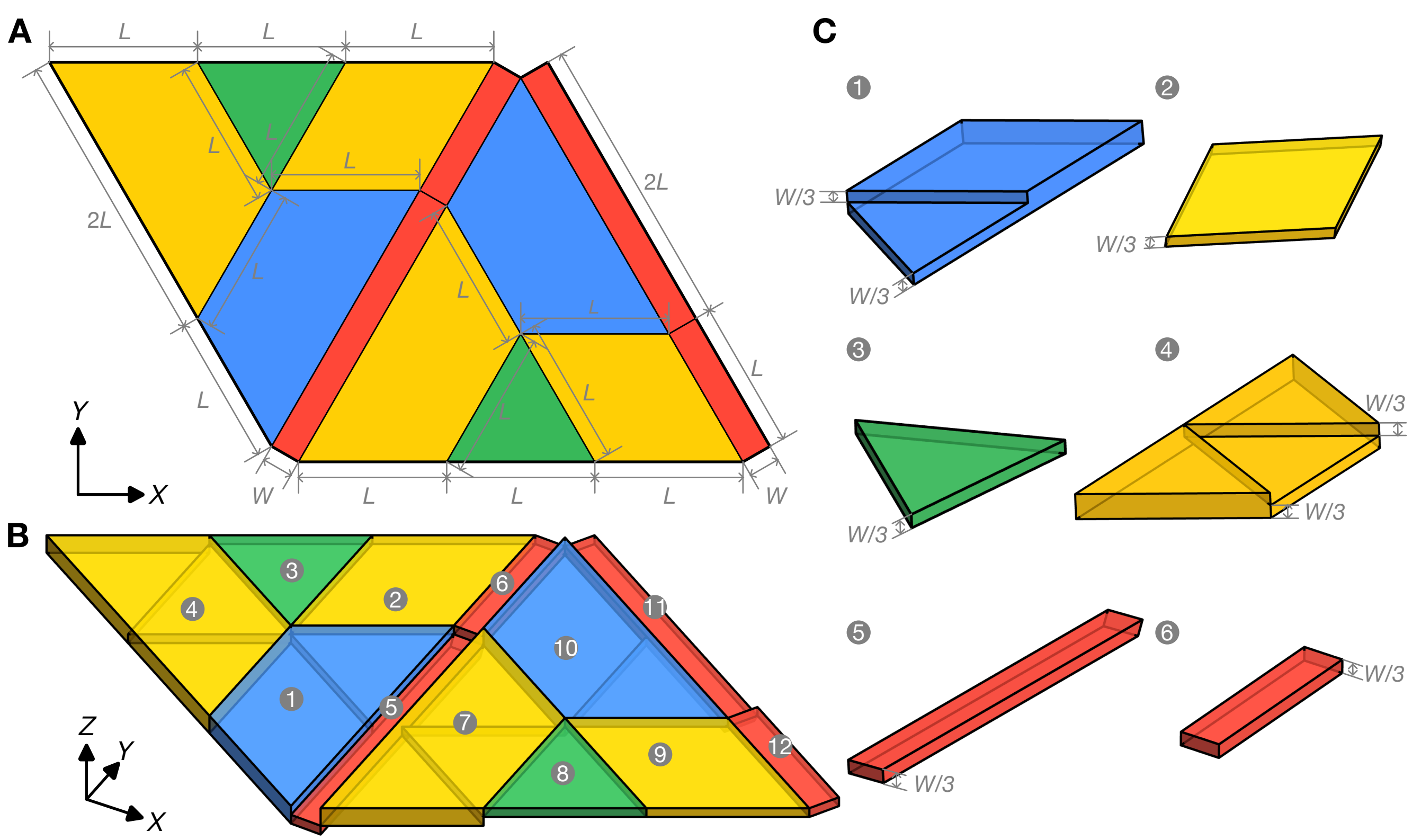

Fig. S4. Geometric dimensions of the zero-thickness and thick-panel origami underlying the prototype shown in Fig. 5 of the main text. (A) Crease pattern of a zero-thickness unit cell. The crease length L and the width W of the inserted rectangular panels are design parameters. In the prototype, $L = 50\mathrm{mm}$ and $W = 10.5\mathrm{mm}$. (B) Unit cell after thickening. Each panel is labeled by an index. Panels 7–12 are congruent to or mirror symmetric to panels 1–6. (C) Panel thicknesses for panels 1–6. The thickness of each panel (corresponding to the link length in the offset-hinge method) is determined by W so that the top and bottom surfaces coincide exactly in the flat-folded state. In the prototype, since $W = 10.5\mathrm{mm}$, the panel thicknesses are given by $W/3 = 3.5\mathrm{mm}$ or its integer multiples.

Movie S1. **Visualization of the adjacent-fold-angle relation and the corresponding folding motion for several values of (θ^0, θ^1), arranged in a grid over the (θ^0, θ^1)-space. The sector angles are taken as $\theta^0, \theta^1 \in \{12.5^\circ, 38.3333^\circ, 64.1667^\circ, 90^\circ, 115.833^\circ, 141.667^\circ, 167.5^\circ\}$ with mode assignment $\sigma = 1$, excluding the singular cases where $\theta^1 = \pi - \theta^0$. The folding motion is generated by varying the initial fold angle ρ_0 linearly at a constant rate. At each step, (ρ^0, ρ^1) and (ρ^0, ρ^3) corresponding to the folded state are plotted on the graph.**

Movie S2. **Visualization of the adjacent-fold-angle relation and the corresponding folding motion for several values of (θ^0, θ^1), arranged in a grid over the (θ^0, θ^1)-space. The sector angles are taken as $\theta^0, \theta^1 \in \{12.5^\circ, 38.3333^\circ, 64.1667^\circ, 90^\circ, 115.833^\circ, 141.667^\circ, 167.5^\circ\}$ with mode assignment $\sigma = -1$, excluding the singular cases where $\theta^1 = \theta^0$. The folding motion is generated by varying the initial fold angle ρ_0 linearly at a constant rate. At each step, (ρ^0, ρ^1) corresponding to the folded state is plotted on the graph.**

Movie S3. **Folding motion of the strip shown in Fig. 3(A) of the main text. The motion is generated by varying the initial fold angle ρ_0 according to $\rho_0(t_0) = 2\arctan(p^{-t_0})$, where $t_0 \in [-8, 24]$ and $p \approx 0.4875$. The corresponding cobweb plot, orbit plot, and sigmoid function, along with its 10–90% transition width at each time step, are also shown.**

Movie S4. **Folding motion of the strip shown in Fig. 3(B) of the main text. The motion is generated by varying the initial fold angle ρ_0 linearly at a constant rate. The evolution of the corresponding cobweb plot and phase-space trajectory at each time step is also shown.**

Movie S5. **Folding motion of the strip shown in Fig. 3(C) of the main text. The motion is generated by varying the initial fold angle ρ_0 linearly at a constant rate. The evolution of the corresponding cobweb plot and phase-space trajectory at each time step is also shown.**

Movie S6. **Folding motion of the strip shown in Fig. 3(D) of the main text. The motion is generated by varying the initial fold angle ρ_0 according to $\rho_0(t_0) = -2\arctan(p^{-t_0})$, where $t_0 \in [-7, 17]$ and $p = 3$. The corresponding cobweb plot, orbit plot, and sigmoid function, along with its 10–90% transition width at each time step, are also shown.**

Movie S7. **Folding motion of the strip shown in Fig. 3(E) of the main text. The motion is generated by varying the initial fold angle ρ_0 according to $\rho_0(t_0) = -2\arctan(p^{-t_0})$, where $t_0 \in [-7, 13]$ and $p = 4$. The corresponding cobweb plot, orbit plot, and sigmoid function, along with its 10–90% transition width at each time step, are also shown.**

Movie S8. **Folding motion of the strip shown in Fig. 3(F) of the main text. The motion is generated by varying the initial fold angle ρ_0 according to $\rho_0(t_0) = -2\arctan(p^{-t_0})$, where $t_0 \in [-7, 13]$ and $p = 4$. The corresponding cobweb plot, orbit plot, and sigmoid function, along with its 10–90% transition width at each time step, are also shown.**

Movie S9. **Folding motion of the strip shown in Fig.4(D) of the main text. The motion is generated by varying the initial fold angle ρ_0 according to $\rho_0(t_0) = -2\arctan(p^{-t_0})$, where $t_0 \in [-7, 32]$ and $p = 4$.**

Movie S10. **Folding motion of the strip shown in Fig.4(E) of the main text. The motion is generated by varying the initial fold angle ρ_0 linearly at a constant rate.**

Movie S11. **Folding motion of a strip that deploys along a Hilbert-curve-like polyline within a confined maze-like environment. The right panel shows the top view of the folding motion together with the target polyline (red). The motion is generated by varying the initial fold angle ρ_0 according to $\rho_0(t_0) = -2\arctan(p^{-t_0})$, where $t_0 \in [-7, 395]$ and $p = 4$.**

Movie S12. **Folding motion of the thickness accommodated strip using the method shown in Fig.5(A) of the main text.**

Movie S13. **Domino-like sequential deployment of the 3D-printed physical prototype shown in Fig.5(D) of the main text. The prototype is initially placed in a flat-folded state on a planar surface, with the soft edges that should deploy first positioned on the upper side. As the upper panel is lifted, the structure undergoes sequential, one-degree-of-freedom deployment under gravity, until reaching the fully deployed state. In the deployed state, the bottom crease corresponds to a soft edge that should be folded first during stowage. Stowage is triggered when the prototype is lowered and contacts the planar surface. The structure then**

returns to the flat-folded state in the reverse order of deployment, recovering its original flat-folded state. The video shows multiple repetitions of this deployment–stowage cycle.

Movie S14. Demonstration of the stiffness and softness of the edge creases of the prototype in the deployed state.

References

1. TC Hull, *Origametry: mathematical methods in paper folding.* (Cambridge University Press, Cambridge, UK), (2020).
2. R Foschi, TC Hull, JS Ku, Explicit kinematic equations for degree-4 rigid origami vertices, Euclidean and non-Euclidean. *Phys. Rev. E* **106**, 055001 (2022).
3. C Hoberman, Reversibly expandable three-dimensional structure (1988) U.S. Patent 4,780,344.
4. Y Chen, R Peng, Z You, Origami of thick panels. *Science* **349**, 396–400 (2015).
5. X Zhang, Y Chen, Mobile assemblies of bennett linkages from four-crease origami patterns. *Proc. Royal Soc. A: Math. Phys. Eng. Sci.* **474**, 20170621 (2018).
6. RJ Lang, KA Tolman, EB Crampton, SP Magleby, LL Howell, A review of thickness-accommodation techniques in origami-inspired engineering. *Appl. Mech. Rev.* **70**, 010805 (2018).
